# Compensatory Interplay Between Clarin‐1 and Clarin‐2 Deafness‐Associated Proteins Governs Phenotypic Variability in Hearing

**DOI:** 10.1002/advs.202521853

**Published:** 2026-01-22

**Authors:** Maureen Wentling, Aïda Yakhlef Sanchez, Nicolas Thelen, Müge Senarisoy, Maria Hogg, Steven Condamine, Andrea Lelli, Emilia Wysocka, Pranav Patni, Sandrine Vitry, Kerem Yasin Yildizhan, Sébastien Le Gal, Sylvie Nouaille, Michael R. Bowl, Marc Thiry, Didier Dulon, Sedigheh Delmaghani, Aziz El‐Amraoui

**Affiliations:** ^1^ Institut Pasteur Institut De L'audition, AP‐HP, INSERM U1335, CNRS, Fondation Pour l'Audition, IHU reConnect, Progressive Sensory Disorders, Pathophysiology and Therapy Université Paris Cité Paris France; ^2^ Collège Doctoral ED515 Sorbonne Université Paris France; ^3^ Collège Doctoral ED158 Sorbonne Université Paris France; ^4^ Cellular and Tissular Biology, GIGA‐Neurosciences University of Liège Liège Belgium; ^5^ Laboratoire De Neurophysiologie de La Synapse Auditive Institut De L'audition and Université de Bordeaux Bordeaux France; ^6^ Institut Pasteur Institut De L'audition, AP‐HP, INSERM, CNRS, Fondation Pour l'Audition, IHU reConnect Auditory Therapies Innovation Laboratory Université Paris Cité Paris France; ^7^ UCL Ear Institute University College London London UK

**Keywords:** Clarin‐1 and Clarin‐2, hearing phenotypic variability, ion homeostasis, mechanoelectrical transduction, synaptopathy, Usher syndrome type III

## Abstract

Usher syndrome type III (USH3) is a genetic disorder characterized by progressive, post‐lingual hearing loss, variable vestibular dysfunction, and onset of retinitis pigmentosa. USH3 is caused by mutations in *CLRN1*, which encodes clarin‐1, a tetraspanin‐like protein. Mutations in *CLRN2*, which encodes the related protein clarin‐2, are also implicated in progressive, non‐syndromic hearing loss in both humans and mice. USH3 patients show considerable phenotypic variability, even among individuals with the same mutation. This variability may result from environmental factors or interactions with other inner ear genes, such as *CLRN2*. To investigate the functional interplay of these genes, we generated *Clrn1* ^–^^/−^ *Clrn2* ^−/−^ double knockout mice. RNA‐sequencing and functional/physiological analyses revealed that clarin‐1 and clarin‐2 jointly regulate mechanoelectrical transduction, ionic homeostasis, and synaptic organization. Their combined loss leads to more severe hearing phenotype compared to *Clrn1* ^−/−^ and *Clrn2* ^−/−^ mice, which reveals a functional compensation between them. *CLRN2* variants may exacerbate hearing loss in USH3 patients, supporting inclusion of *CLRN2* in genetic screening. By revealing a functional, compensatory interplay between clarin‐1 and clarin‐2, this study reframes *CLRN1‐*associated deafness as a network‐dependent disorder and provides a mechanistic basis for genetic stratification and therapeutic directions in USH3 and related sensorineural hearing loss.

## Graphical abstract

Functional compensation between clarin‐1 and clarin‐2 in cochlear hair cells. Hearing loss associated with CLRN1 mutations shows striking phenotypic variability; however, the underlying mechanisms remain poorly understood. This study reveals that clarin‐1 and clarin‐2 function cooperatively in cochlear hair cells to sustain mechanoelectrical transduction, ionic homeostasis, and synaptic stability. Partial compensation between the two proteins buffers auditory function, whereas their combined loss triggers severe auditory failure, redefining clarin‐associated deafness as a network‐dependent disorder.

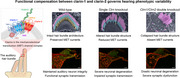

## 1 Introduction

Hearing loss is among the most prevalent sensory disorders worldwide, affecting over 5% of the global population (World Health Organization, 2021). It spans all ages and etiologies, from congenital forms—present in approximately 1 in 500 newborns—to age‐related hearing loss, which affects nearly two‐thirds of individuals over 70 [1, 2, 3]. The consequences are substantial, including impaired communication, social withdrawal, cognitive decline, and depression [4]. Despite its prevalence and impact, there are no curative treatments for sensorineural hearing loss. Hearing aids and cochlear implants offer partial restoration for some individuals, but biological therapies to prevent, halt, or reverse inner ear damage remain elusive.

Inherited forms of deafness account for more than half of congenital and early‐onset cases. These disorders are characterized by considerable genetic heterogeneity [3, 5] and variable clinical presentation, even among individuals with the same causal mutation. One illustrative example is Usher syndrome (USH), an autosomal recessive disorder, and the number one cause of deaf‐blindness. USH is caused by 9 distinct genes with 3 clinical subtypes (USH1, USH2, and USH3) [6, 7, 8, 9]. Usher syndrome type III patients have post‐lingual and variable progressive hearing loss, with variable vestibular deficits, and variable onset of retinitis pigmentosa. Clarin‐1, the causative gene for USH3, belongs to the clarin tetraspanin‐like family, which contains 3 members: clarin‐1, clarin‐2, and clarin‐3. All three genes encode a 4 transmembrane domain protein, with 60% shared sequence homology; however, only clarin‐1 and clarin‐2 are expressed in the inner ear. Clarin‐2 was first identified as a putative deafness gene through a UK Biobank study that correlated single‐nucleotide polymorphisms at the *CLRN2* locus with self‐reported hearing difficulties [10]. Recently, mutations in *CLRN2* were reported in consanguineous families suffering from early onset, moderate to profound sensorineural hearing loss, and *CLRN2* was identified definitively as a deafness gene, DFNB117 [11, 12, 13].

Clarin‐1 and clarin‐2 are expressed embryonically in the inner ear and are required for normal audition [10, 14, 15, 16]. Mice lacking clarin‐1 constitutively (*Clrn1* ^−/−^) are profoundly deaf at hearing onset and have severely fragmented hair bundles with missing stereocilia [15, 16]. *Clrn1* ^−/−^ mice display increased inward calcium currents with reduced calcium‐mediated exocytosis [16]. Early loss of primary auditory neurons and no detectable electrically evoked brainstem responses were also noted in these mice [16]. Mice lacking clarin‐2 have an early onset and progressive hearing loss [10]. In *Clrn2* ^−/−^ mice, unlike *Clrn1* ^−/−^ mice, the hair bundles form the characteristic V‐shape; however, the short row stereocilia progressively degenerate after hearing onset [10]. These losses were correlated with reduced mechanoelectrical transduction (MET) currents [10]. MET function is not only vital for sound transmission, but also for the proper development and maintenance of primary auditory neurons [17, 18, 19].

Usher syndrome type III patients experience a wide range of phenotypic variability, even among patients with the same genetic mutations, particularly in hearing loss progression and severity [20, 21]. This phenotypic auditory variability in USH3 may be due to environmental factors or even the genetic complexity of *CLRN1* itself, which has multiple splice variants [22]. However, we suggest that interactions with additional genes in the inner ear, such as clarin‐2, may play a role in this phenotypic variability.

To answer this question and disentangle the key role(s) of each clarin in the auditory system, we employed a comprehensive approach integrating constitutive and conditional single and double clarin knockout mouse models, electrophysiological recordings, ultrastructural imaging, and transcriptomic profiling. By dissecting the individual and combined roles of clarin‐1 and clarin‐2 across multiple levels of cochlear biology, we define a compensatory relationship between these proteins that is essential for MET integrity, hair cell function, synaptic organization, and neuronal survival. Our findings offer mechanistic insight into the variable phenotypes observed in USH3 patients and highlight network interactions in inherited sensory disorders.

## 2 Results

### 2.1 Clarin‐1 and Clarin‐2 Play Compensatory Roles in Mechanoelectrical Transduction

To investigate the functional relationship between clarin‐1 and clarin‐2, we generated clarin‐1/clarin‐2 total knockout (*Clrn1* ^−/−^ *Clrn2* ^−/−^) mice by crossing *Clrn1* ^−/−^ and *Clrn2* ^−/−^ mice. Hearing function was assessed at P21 in *Clrn1* ^−/−^ *Clrn2* ^−/−^ mice by auditory brainstem response (ABR). No detectable electrical signal (ABR waves) could be recorded at any frequency tested (5 – 40 kHz), with sound stimulation tested up to 100 dB SPL (Figure S1A). Outer hair cell (OHC) function was also assessed at P21 by distortion product otoacoustic emission (DPOAE) recording. No DPOAE responses could be detected at any frequency tested from 8 to 32 kHz (16 kHz shown; Figure S1B). These results are consistent with *Clrn1* ^−/−^ mice, who are profoundly deaf at P21 and do not have recordable DPOAE responses [16].

**FIGURE 1:**
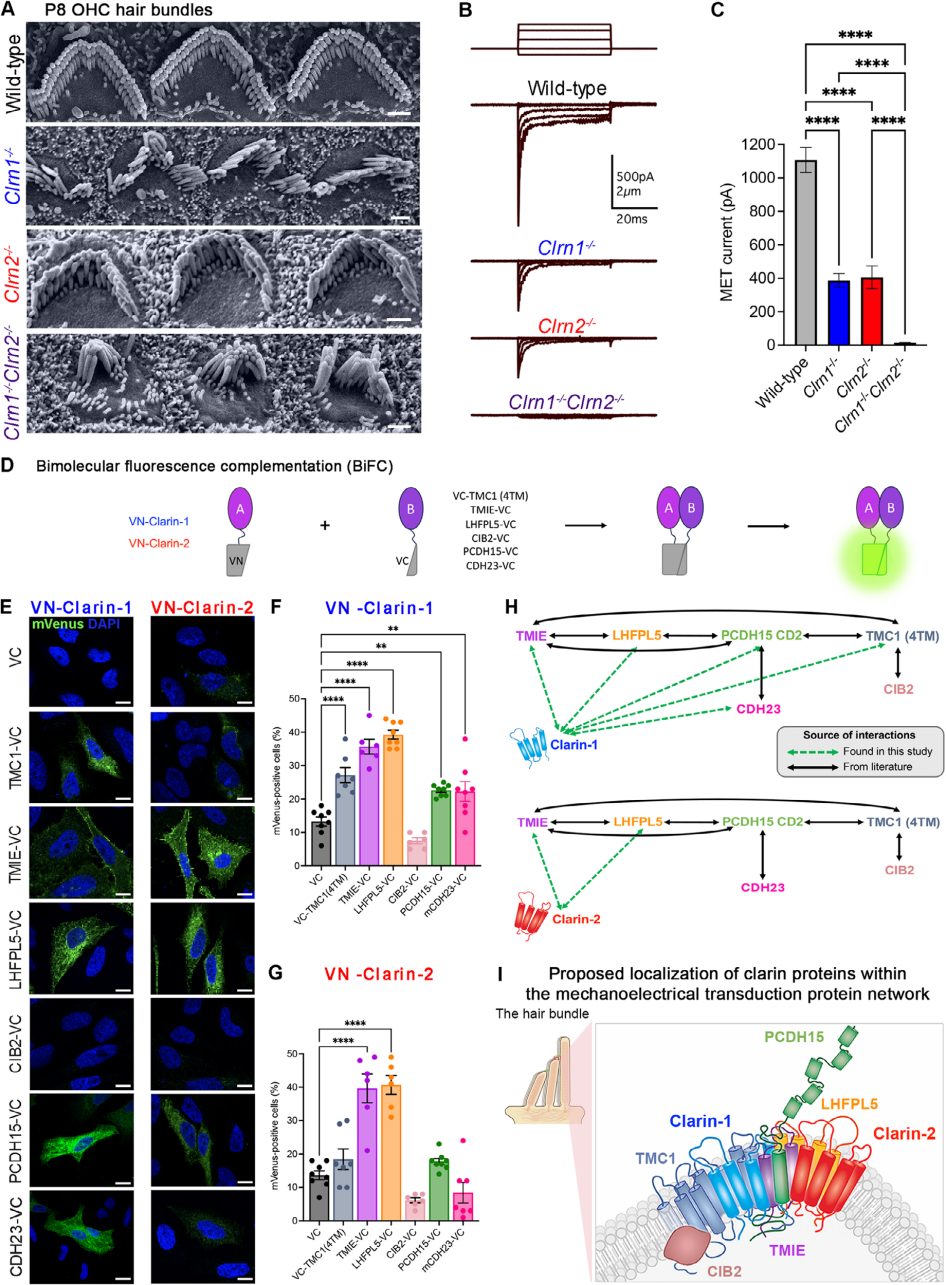
Clarin‐1 and clarin‐2 play a vital and compensatory role in mechanoelectrical transduction. (A) Scanning electron microscopy (SEM) of OHC hair bundles in wild‐type, *Clrn1* ^−/−^, *Clrn2* ^−/−^, and *Clrn1* ^−/−^ *Clrn2* ^−/−^ mice at P8 at the middle turn of the cochlea (scale bar = 1 µm). (B) Sample MET current traces in wild‐type, *Clrn1* ^−/−^, *Clrn2* ^−/−^, and *Clrn1* ^−/−^ *Clrn2* ^−/−^ mice at P6‐P7 from apical outer hair cells. Bundles were mechanically stimulated in steps, and current was held at ‐80 mV. (C) Quantification of maximal MET current amplitudes in wild‐type, *Clrn1* ^−/−^, *Clrn2* ^−/−^, and *Clrn1* ^−/−^ *Clrn2* ^−/−^ mice. *n* = 10–20 hair bundles in 3 mice per genotype with 5 recordings per hair bundle. Data are presented as mean ± SEM and were analyzed using one‐way ANOVA with Tukey's multiple comparisons test. (D) Schematic of BiFC assay. Non‐fluorescent N‐terminal of mVenus (1‐172aa) is conjugated to clarin‐1 or clain‐2, while MET proteins are conjugated to the non‐fluorescent C‐terminal of mVenus (155‐238aa). Proteins are co‐expressed in HeLa cells; if the conjugated proteins interact, the complementation of mVenus occurs, and the protein fluoresces at 528 nm. (E) mVenus fluorescence in transfected HeLa cells with clarin‐1 or clarin‐2 and the various members of the MET machinery, namely TMC1, TMIE, LHFPL5, CIB2, PCDH15, CDH23, and USH1C (scale bar = 10 µm). (F) Quantification of the percent of mVenus‐positive transfected cells for MET machinery with clarin‐1. As compared to control, clarin‐1 interacts with TMC1, TMIE, LHFPL5, PCDH15, CDH23, and USH1C. *n* = 6–8 biological replicates; data represented as mean ± SEM; analyzed by one‐way ANOVA with Dunnett's multiple comparisons test. (G) Quantification of the percent of mVenus‐positive transfected cells for MET machinery with clarin‐2. As compared to control, clarin‐1 interacts with TMIE, LHFPL5, and USH1C. *n* = 6–8 biological replicates; data represented as mean ± SEM; analyzed by one‐way ANOVA with Dunnett's multiple comparisons test. (H) Clarin interactome with MET machinery. (I) Schematic of the proposed composition of MET machinery.

Both *Clrn1* and *Clrn2* are expressed in inner and outer hair cells of the cochlea, where they contribute to the organization and stability of stereocilia bundles [10, 15, 16, 23]. Given the early auditory deficits observed in clarin mutants, we next examined whether hair bundle architecture shows genotype‐dependent alterations by assessing stereocilia morphology in *Clrn1* ^−/−^, *Clrn2* ^−/−^, and *Clrn*1^−/−^ *Clrn2* ^−/−^ mice from early postnatal stages onward (Figure 1A,C; Figure S1C,D). Across OHCs and IHCs, we observed a graded spectrum of structural disruption of hair bundle integrity according to *Clrn* genotype (Figure S1C,D). Despite loss of short row stereocilia, *Clrn2* ^−/−^ OHCs V‐shaped bundles and IHCs U‐shaped bundles remained largely intact, whereas *Clrn1* ^−/−^ hair cells displayed moderate disorganization, including misaligned rows and early fragmentation (Figure S1C,D; see also [10, 16]). Strikingly, *Clrn1* ^−/−^ *Clrn2* ^−/−^ bundles exhibited profound collapse, with severely fragmented, misoriented, and truncated stereocilia and near‐complete loss of coherent V‐ or U‐shaped architecture (cf. quantitative data in Figure s1D). Together, these findings demonstrate that clarin‐1 is essential for bundle integrity and that clarin‐2 provides partial compensatory support, with dual loss precipitating a drastic bundle collapse.

Hair bundle morphology is altered in *Clrn1* ^−/−^ mice, therefore [16], we assessed the hair bundle morphology of *Clrn1* ^−/−^, *Clrn2* ^−/−^, and *Clrn*1^−/−^ *Clrn2* ^−/−^ mice from P8 – P60 (Figure 1A, Figure S1E). *Clrn1* ^−/−^ *Clrn2* ^−/−^ mice have highly fragmented and disorganized hair bundles with missing stereocilia regardless of row height. This represents an exacerbation of the fragmentation in single *Clrn1* ^−/−^ mice as well as the normal OHC bundles found in *Clrn2* ^−/−^ mice (Figure 1A, S1E).

We next investigated the role of clarin‐1 and clarin‐2 in mechanoelectrical transduction. Hair bundles of voltage‐clamped OHCs were mechanically stimulated, and whole‐cell MET currents were recorded at P6‐P7 in wild‐type, *Clrn1* ^−/−^, *Clrn2* ^−/−^, and *Clrn1* ^−/−^ *Clrn2* ^−/−^ mice (Figure 1B,C). Hair cells with the most intact hair bundles that displaced properly were chosen for analysis. We found that *Clrn1* ^−/−^ *Clrn2* ^−/−^ mice had no recordable MET currents, and as expected, *Clrn1* ^−/−^ and *Clrn2* ^−/−^ mice had reduced MET currents (Figure 1C). These data strengthen the hypothesis that clarin‐1 and clarin‐2 play a role in either MET function or maintenance and demonstrate a compensatory role between clarin‐1 and clarin‐2 in mechanoelectrical transduction.

To decipher the molecular pathways implicated in clarin‐mediated hearing loss, whole organs of Corti were dissected from P21 wild‐type, *Clrn1* ^−/−^, *Clrn2* ^−/−^, and *Clrn1* ^−/−^ *Clrn2* ^−/−^ mice to perform RNA‐sequencing. Principal component analysis (PCA) segregated each genotype nicely, with no outliers found among the replicates (Figure S2A). Differential analysis of each clarin mutant relative to wild‐type was performed using a 1.5‐fold change cutoff, with significance set at q‐value ≤ 0.05 to control for false discoveries associated with multiple hypothesis testing (Tables S1–S3). Relative to wild‐type, there were 525 over‐expressed and 484 under‐expressed genes in *Clrn1* ^−/−^ mice, 152 over‐expressed and 171 under‐expressed genes in *Clrn2* ^−/−^ mice, and 1020 over‐expressed and 644 under‐expressed genes *Clrn1* ^−/−^ *Clrn2* ^−/−^ mice (Figure S2A). *Clrn1* ^−/−^ mice showed approximately 3.5× more upregulated genes (525 vs. 152) and 2.9× more downregulated genes (494 vs. 171) than *Clrn2^−/−^* mice. This observation aligns with the more severe phenotype in *Clrn1* ^−/−^ mice, indicating that *Clrn1* regulates a broader functional repertoire than *Clrn2*.

Interestingly, transcriptomic profiling at P21 revealed that 35 hearing‐related genes were significantly dysregulated in clarin‐deficient mice, including 14 genes previously implicated in mechanoelectrical transduction (MET) and hair bundle–tectorial membrane coupling (Figure S2B). The majority of these transcripts showed increased expression, consistent with a compensatory transcriptional response to impaired MET function in the absence of clarin‐1 and clarin‐2. To independently assess these changes, we performed targeted quantitative real‐time PCR (qRT‐PCR) analysis of the 14 MET‐related genes, comparing P21 organ of Corti of *Clrn1* ^−/−^ *Clrn2* ^−/−^ mice relative to wild‐type (*n* = 3 per group) (Figure S2C). qRT‐PCR confirmed the significant upregulation of Pcdh15, Diaph1, Espnl, Otog, Ptprq, Tmc1, Col11a1, and Ceacam16. Tectb, Otogl, Ush1c, Strc, *Espn*, and Pdzd7, which exhibited more modest fold changes in RNA‐seq, were not significantly different from wild‐type via qRT‐PCR, likely reflecting expression changes near the sensitivity threshold of targeted assays. Together, these data support a coordinated, clarin‐dependent transcriptional response at P21 affecting key molecular pathways required for MET and hair bundle integrity.

Given these alterations in the expression of MET complex genes and MET function, we hypothesized clarin‐1 and clarin‐2 act as accessory proteins in the MET complex. TMC1 and PCDH15, with TMIE (Transmembrane Inner Ear), LHFPL5 (LHFPL Tetraspan Subfamily Member 5), and CIB2 (Calcium‐ and Integrin‐Binding protein 2), make up the known components of the MET complex [24, 25, 26]. A high‐throughput study of human protein interactions first identified clarin‐1 as interacting with TMIE [27]. To test this hypothesis, we studied the clarin‐1 and clarin‐2 MET protein interactome in HeLa cells using bimolecular fluorescence complementation (BiFC) (Figure 1D, E). We tested the possible interaction of clarin‐1 and clarin‐2 specifically with four transmembrane (TM) domains of TMC1 (TM 3–6; amino acids 348–556), full length TMIE, LHFPL5, CIB2, and the tip‐link proteins PCDH15 (extracellular cadherin (EC) 11, extracellular linker (EL), TM, cytoplasmic region and CD2 isoform; amino acids 1–30 and 1152–1790), CDH23 (EC 26–27, TM, cytoplasmic region containing exon 68; amino acids 2729–3354), and full length USH1C. The tip‐link protein, PCDH15, has three isoforms (CD1, CD2, and CD3); however, only the CD2 isoform is indispensable for MET function and hearing [28]. CDH23 containing exon 68 is essential for maintaining tip‐link stability [29]. The previously established interactions of clarin‐1 with TMIE [27] and USH1C [16] were validated (Figure 1E,F,H), and clarin‐1 was also found to interact with TMC1, LHFPL5, PCDH15, CDH23, but not CIB2 (Figure 1E,F,H,I). Clarin‐2 was also found to interact with TMIE, LHFPL5, and USH1C (Figure 1E,G,H). Known and novel interactions within MET machinery, along with clarin‐1 and clarin‐2 are summarized in Figure 1H,I. Taken together, these data implicate clarin‐1 and clarin‐2 as playing partially overlapping and complementary roles as potential accessory proteins in the MET channel protein complex.

#### 2.1.1 Clarin‐1 and Clarin‐2 are Required for Ionic Homeostasis

**FIGURE 2:**
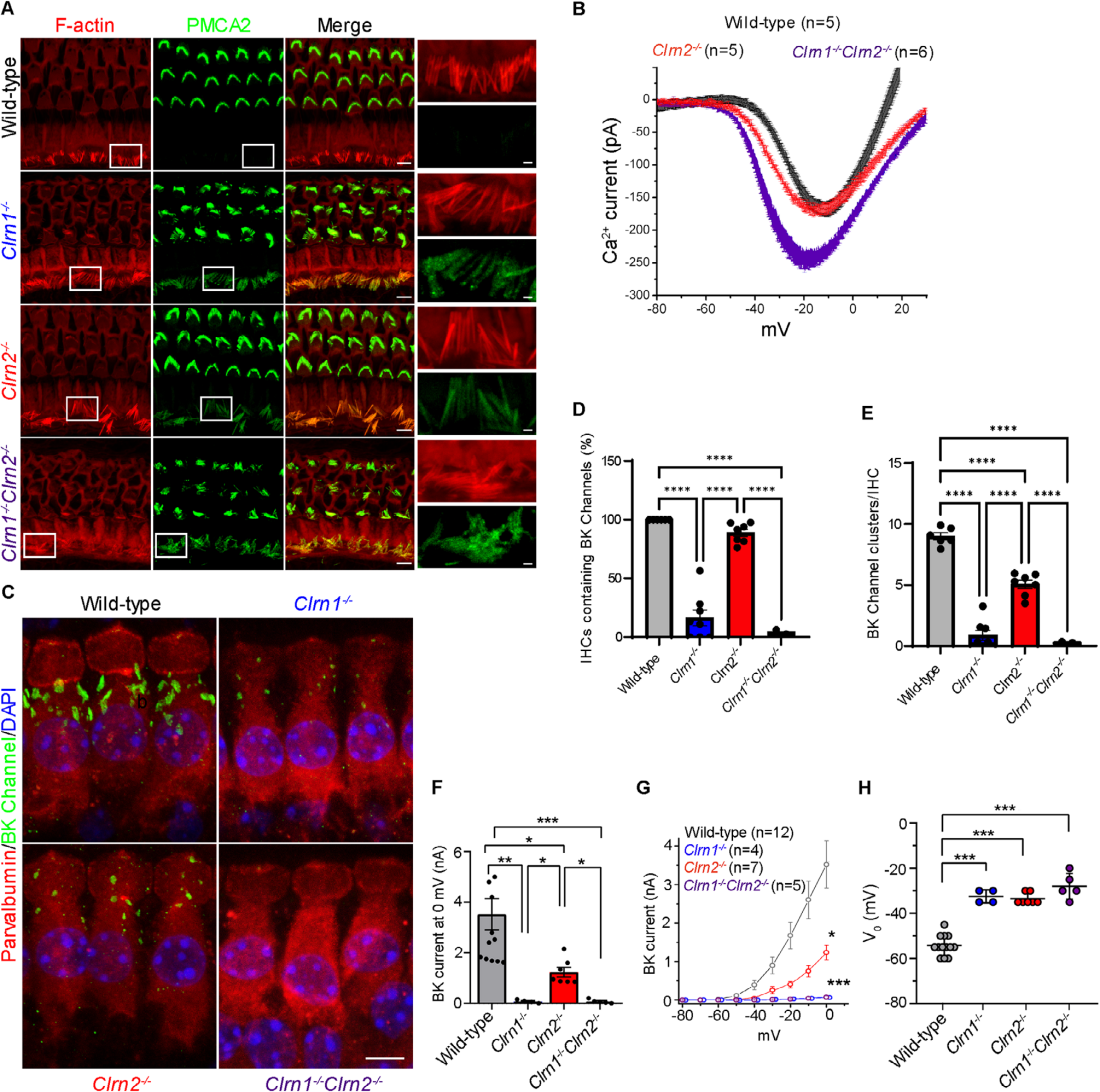
Clarin‐1 and clarin‐2 are required for ionic homeostasis in hair cells. (A) F‐actin (red) and PMCA2 (green) immunostaining of IHC and OHC hair bundles in wild‐type, *Clrn1* ^−/−^, *Clrn2* ^−/−^, and *Clrn1* ^−/−^ *Clrn2* ^−/−^ mice at P21 at the apical turn of the cochlea, inset shows magnified IHCs (scale bar = 5 µm, inset scale bar = 1 µm). (B) Comparative Ca^2+^ currents in IHCs of P20‐P25 Clarin mutants: Whole‐cell patch‐clamped IHCs were subjected to a voltage ramp stimulation from −90 to +30 mV in 120 ms, giving a slope change in voltage of 1 mV/ms. IHCs from *Clrn1* ^−/−^ *Clrn2* ^−/−^ mice displayed larger peak Ca^2+^ current amplitudes (Kruskal‐Wallis test with multiple comparison) and larger voltage‐shift in their activation curve as compared to *Clrn2* ^−/−^ and wild‐type mice (V_1/2_ shift; Kruskal‐Wallis test with multiple comparison). (C) Parvalbumin (red), BK channel (green), and DAPI (blue) immunostaining in IHCs of wild‐type, *Clrn1* ^−/−^, *Clrn2* ^−/−^, and *Clrn1* ^−/−^ *Clrn2* ^−/−^ mice at P21 at the middle turn of the cochlea (scale bar = 5 µm). (D,E) Quantification of IHCs containing BK channel clusters. (F) Quantification of BK channel clusters per IHC (in the IHCs that contained BK channels). Wild‐type *n* = 6, *Clrn1* ^−/−^ *n* = 8, *Clrn2* ^−/−^ *n* = 7, and *Clrn1* ^−/−^ *Clrn2* ^−/−^ *n* = 5, with minimum 30 hair cells analyzed per *n*. Data are presented as mean ± SEM and were analyzed using one‐way ANOVA with Tukey's multiple comparisons test. (F, G) Comparative BK currents in IHCs of (P18‐P24) Wild‐type and clarin‐deficient mice. IHCs were voltage‐clamped at ‐70 mV and depolarized for 10 ms at various membrane potentials. The fast‐activating outward BK currents (IK_f_) are largely reduced in IHCs of *Clrn2* ^−/−^ mice and were completely absent in IHCs of *Clrn1* ^−/−^ and *Clrn1* ^−/−^ *Clrn2* ^−/−^ mice. The amplitudes of BK currents were compared at ‐20 mV in (F) and plotted as a function of voltage (I/V curve) in (G). Data are presented as mean ± SEM and were analyzed using Kruskal‐Wallis test with multiple comparison (F) and two‐way ANOVA with Tukey's multiple comparisons test (G). (H) With KCl‐based recording‐pipette solutions, IHCs' average resting membrane potential (measured as the 0 current potential) was found significantly depolarized in clarin‐mutant mice as compared to wild‐type mice. Wild‐type V_0_ = – 54 ± 4 mV (*n* = 13); *Clrn1* ^−/−^ V_0_ = ‐32 ± 3 mV (*n* = 4); *Clrn2* ^−/−^ V_0_ = ‐34 ± 2 mV (*n* = 7); *Clrn1* ^−/−^ *Clrn2* ^−/−^ V_0_ = ‐30 ± 6 mV (*n* = 5). Data are presented as mean ± SD and were analyzed using one‐way ANOVA with Tukey's multiple comparisons test.

We next investigated whether clarin‐1 and clarin‐2 play a role in ionic homeostasis. We first assessed the expression of plasma membrane calcium ATPase (PMCA2) in mature hair cells of clarin‐deficient mice. PMCA2 is the main regulator of intrastereociliar calcium concentration and is vital to proper actin polymerization and MET channel function and adaptation [30]. It is highly expressed in the stereocilia of OHCs, and its expression is much weaker in IHCs and down‐regulated upon hearing onset [30]. We found that PMCA2 expression was up‐regulated in the IHCs of all clarin‐deficient mice compared to wild‐type mice in the apical turn of the cochlea, but not in the middle and basal turns at P21 (Figure 2A). Moreover, comparative analysis of voltage‐dependent Ca^2+^ currents in IHCs of P20‐25 mice revealed a significant increase in current amplitudes in *Clrn1* ^−/−^ *Clrn2* ^−/−^ mice as compared to wild‐type and *Clrn2* ^−/−^ (250 ± 33 pA as compared to 170 ± 8 pA and 176 ± 7 pA, *p* = 0.02 and *p* = 0.01, Kruskal‐Wallis multiple comparison test, Figure 2B). The voltage‐dependance of Ca^2+^ currents in *Clrn1* ^−/−^ *Clrn2* ^−/−^ mice was affected similarly to *Clrn1* ^−/−^ mice [16], with a half‐voltage activation (V_1/2_) being shifted toward more negative potentials as compared to *Clrn2* ^−/−^ and wild‐type mice (half‐voltage activation V_1/2_ = −34 ± 0.7 mV in *Clrn1* ^−/−^ *Clrn2* ^−/−^, as compared to V_1/2_ = −29 ± 1.5 mV, V_1/2_ = – 26 ± 0.8 mV, in *Clrn2* ^−/−^ and wild‐type mice, respectively; *P* <0.001).

We next assessed the expression of the large conductance voltage‐gated potassium channel, the BK channel, in mature IHCs at P21 (Figure 2C). BK channels are vital to IHC repolarization, controlling their excitability and afferent glutamatergic synaptic transmission. BK channels are concentrated at the apical membrane of IHCs, just below the cuticular plate, called the neck, where they form clusters [31, 32]. BK channel currents become active after the onset of hearing and coincide with MET‐activated, graded action potentials [31, 33]. BK channel distribution in IHCs of *Clrn1* ^−/−^ and *Clrn2* ^−/−^ mice was not altered, and clusters were found normally concentrated at the cuticular plate and along the neck of IHCs (Figure 2C). However, BK channel clusters were much smaller in *Clrn1* ^−/−^ and *Clrn2* ^−/−^ mice, with a more severe reduction in size observed in *Clrn1* ^−/−^ mice. Additionally, not all *Clrn1* ^−/−^ IHCs expressed BK channels. Indeed, 37.5% of *Clrn1* ^−/−^ mice had no BK channel expression in their IHCs; however, all *Clrn2* ^−/−^ mice analyzed had BK channel expression in at least one IHC (Figure 2D). Of IHCs expressing BK channel clusters, *Clrn1* ^−/−^ and *Clrn2* ^−/−^ IHCs also had greatly reduced numbers of clusters relative to wild‐type, with a more severe phenotype found in *Clrn1* ^−/−^ mice (Figure 2E). Strikingly, *Clrn1* ^−/−^ *Clrn2* ^−/−^ mice had no BK channel expression in their IHCs, demonstrating a compensatory effect between clarin‐1 and clarin‐2 in potassium homeostasis. Furthermore, the resting membrane potentials of all IHCs lacking clarin‐1 and/or clarin‐2 were significantly depolarized relative to wild‐type control, indicating a baseline physiological stress of the hair cells due to ionic imbalance (Figure 2H).

We then recorded whole‐cell K^+^ currents in IHCs from wild‐type and mutant adult mice (P18‐P24) in physiological Na^+^/ K^+^ conditions during a brief 10 ms voltage‐step protocol to isolate BK currents. To minimize possible contamination by IK_s_, the amplitudes of the outward BK currents were measured 2 ms after the start of the depolarizing voltage‐steps. *Clrn2* ^−/−^ mice displayed largely reduced BK currents as compared to wild‐type mice (Figure 2F,G). In these IHCs lacking *Clrn2*, the BK conductance showed an apparent 20 mV positive voltage‐shift in its half voltage‐activation (V_1/2_ = −16 ± 2 mV; *n* = 7) as compared to wild‐type (V_1/2_ = −39 ± 2 mV; unpaired *t*‐test, *p*<0.001; Figure 2G). As expected, *Clrn1* ^−/−^ and *Clrn1* ^−/−^ *Clrn2* ^−/−^mice displayed no rapidly activating outward currents (Figure 2G). Overall, these electrophysiological recordings are consistent with the quantification of the BK channel immunoreactivity in IHCs of clarin‐mutant mice, revealing that *Clrn1* is essential for the developmental expression of BK channels in IHCs while *Clrn2* is partially dispensable.

A large category of significantly dysregulated genes in clarin‐mutant mice was those implicated in ionic homeostasis. There was almost equal up‐ and down‐regulation of genes responsible for calcium, potassium, and sodium homeostasis in clarin‐deficient mice relative to wild‐type (Figure S3), indicating an overall cationic imbalance.

To clarify whether these ionic abnormalities originated from hair cell and MET dysfunction, we generated hair cell‐conditional clarin knockout mice: *Clrn1* ^fl/fl^ *Myo15‐cre* ^+/ki^, *Clrn2* ^fl/fl^ *Myo15‐cre* ^+/ki^, and *Clrn1* ^fl/fl^ *Clrn2* ^fl/fl^ *Myo15‐cre* ^+/ki^. These mice lack clarin‐1 and/or clarin‐2 from hair cells starting on postnatal day 3. We first assessed auditory function in these mice by ABR and DPOAE. At P21, *Clrn1* ^fl/fl^ *Myo15‐cre* ^+/ki^ and *Clrn2* ^fl/fl^ *Myo15‐cre* ^+/ki^ mice are moderately to profoundly deaf, while *Clrn1* ^fl/fl^ *Clrn2* ^fl/fl^ *Myo15‐cre* ^+/ki^ mice had no detectable ABR traces at 100 dB SPL of stimulation across all frequencies tested (Figure S4A). By 1 month of age, *Clrn1* ^fl/fl^ *Myo15‐cre* ^+/ki^ and *Clrn2* ^fl/fl^ *Myo15‐cre* ^+/ki^ mice were all profoundly deaf (Figure S4B). As in *Clrn1^−/−^Clrn2^−/−^* mice (Figure S1), DPOAE responses were absent in *Clrn1* ^fl/fl^ *Clrn2* ^fl/fl^ *Myo15‐cre* ^+/ki^ mice at P21 (Figure S4C,D). *Clrn1* ^fl/fl^ *Myo15‐cre* ^+/ki^ mice had reduced DPOAE amplitudes and increased DPOAE thresholds relative to wild‐type (Figure S4C,D). Interestingly, *Clrn2* ^fl/fl^ *Myo15‐cre^+/ki^* mice maintained normal DPOAE responses despite profound loss of hearing by 1 month of age (Figure S4D). Together, these data demonstrate a compensatory role between clarin‐1 and clarin‐2, specifically in hair cells, to mediate hearing, with clarin‐1 playing a more important role in proper OHC function.

**FIGURE 3:**
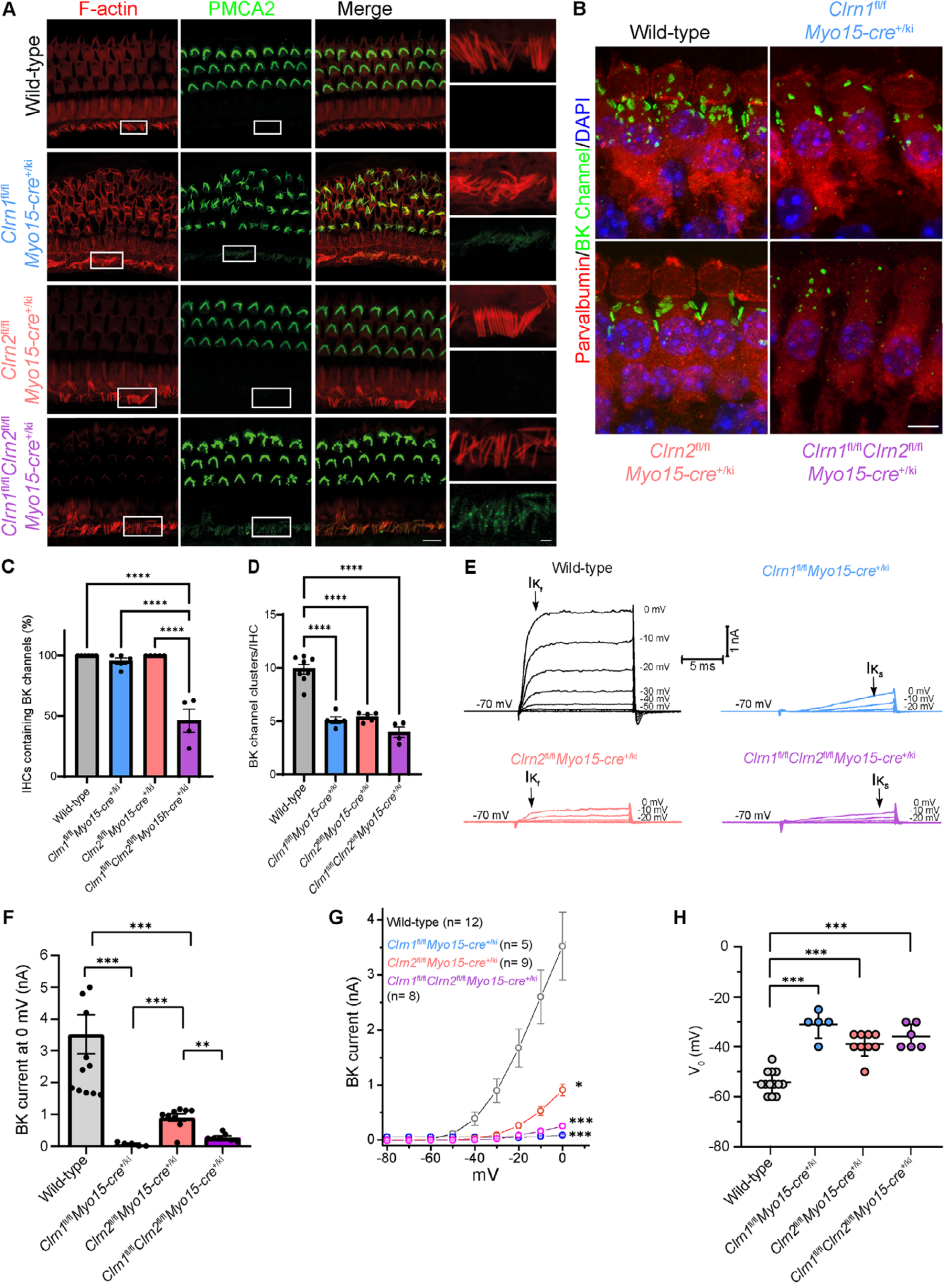
Hair cell‐specific ablation of clarin‐1 and clarin‐2 confirm their role in ionic homeostasis. (A) F‐actin (red) and PMCA2 (green) immunostaining of IHC and OHC hair bundles in wild‐type, *Clrn1* ^fl/fl^ *Myo15‐cre* ^+/ki^, *Clrn2* ^fl/fl^ *Myo15‐cre* ^+/ki^, and *Clrn1* ^fl/fl^ *Clrn2* ^fl/fl^ *Myo15‐cre* ^+/ki^ mice at P21 at the apical turn of the cochlea (scale bar = 5 µm, inset scale bar = 1 µm). (B) Parvalbumin (red), BK channel (green), and DAPI (blue) immunostaining in IHCs of wild‐type, *Clrn1* ^fl/fl^ *Myo15‐cre* ^+/ki^, *Clrn2* ^fl/fl^ *Myo15‐cre* ^+/ki^, and *Clrn1* ^fl/fl^ *Clrn2* ^fl/fl^ *Myo15‐cre* ^+/ki^ mice at P21 at the middle turn of the cochlea (scale bar = 5 µm). (C) Quantification of IHCs containing BK channel clusters (D) Quantification of BK channel clusters per IHC (in the IHCs that contained BK channels). Wild‐type *n* = 6, *Clrn1* ^fl/fl^ *Myo15‐cre* ^+/ki^ and *Clrn2* ^fl/fl^ *Myo15‐cre* ^+/ki^ *n* = 5, *Clrn1* ^fl/fl^ *Clrn2* ^fl/fl^ *Myo15‐cre* ^+/ki^ *n* = 4, with minimum 30 hair cells analyzed per *n*. Data are presented as mean ± SEM and were analyzed using one‐way ANOVA with Tukey's multiple comparisons test. (E‐G) Comparative BK currents in IHCs of (P18‐P24) wild‐type and conditional clarin‐deficient mice. IHCs were voltage‐clamped at ‐70 mV and depolarized for 10 ms at various membrane potentials. The fast‐activating outward BK currents (IK_f_) were largely reduced in IHCs of *Clrn2* ^fl/fl^ *Myo15‐cre* ^+/ki^ mice and were absent in IHCs of *Clrn1* ^fl/fl^ *Myo15‐cre* ^+/ki^ and *Clrn1* ^fl/fl^ *Clrn2* ^fl/fl^ *Myo15‐cre* ^+/ki^ mice (E). The amplitude of BK currents was compared at 0 mV in (F) and analyzed using Kruskal‐Wallis test with multiple comparison. The amplitude of BK currents was plotted as a function of voltage (I/V curve) in (G) and analyzed using two‐way ANOVA with Tukey's multiple comparisons test. To avoid contamination with the slowly activating K^+^ currents (IK_S_), the amplitude of the fast‐activating BK currents was measured 2 ms after the onset of each voltage‐steps. (H) With KCl‐based recording‐pipette solutions, IHCs average resting membrane potential (measured as the 0 current potential) was found significantly depolarized in conditional clarin‐mutant mice as compared to wild‐type mice. Wild‐type: V_0_ = – 54 ± 4 mV (*n* = 13); *Clrn1* ^fl/fl^ *Myo15‐cre* ^+/ki^: V_0_ = ‐32 ± 6 mV (*n* = 5); *Clrn2* ^fl/fl^ *Myo15‐cre* ^+/ki^: V_0_ = ‐39 ± 5 mV (*n* = 9); *Clrn1* ^fl/fl^ *Clrn2* ^fl/fl^ *Myo15‐cre* ^+/ki^: V_0_ = ‐36 ± 5 mV (*n* = 6). Data represented as means ± SD and analyzed using one‐way ANOVA with Tukey's multiple comparisons test.

We then assessed the expression of PMCA2 and BK channels in hair cell‐conditional clarin knockout mice (Figure 3). Unlike in total clarin knockout mice, we found a more modest increase in PMCA2 expression in the IHCs of *Clrn1* ^fl/fl^ *Myo15‐cre* ^+/ki^ and *Clrn1* ^fl/fl^ *Clrn2* ^fl/fl^ *Myo15‐cre* ^+/ki^ mice, with no expression in the IHCs of *Clrn2* ^fl/fl^ *Myo15‐cre* ^+/ki^ mice (Figure 3A). The expression was greater and more uniform in the *Clrn1* ^fl/fl^ *Clrn2* ^fl/fl^ *Myo15‐cre* ^+/ki^ mice relative to *Clrn1* ^fl/fl^ *Myo15‐cre* ^+/ki^ mice, indicating a compensatory role between clarin‐1 and clarin‐2 again.

As in clarin total single knockout mice, BK channel clusters were properly localized to the cuticular plate and along the neck of IHCs in all hair cell‐specific clarin‐1 and clarin‐2 knockout mice (Figure 3B). Unlike in total clarin‐knockout mice, BK channel clusters were present in nearly all inner hair cells of *Clrn1* ^fl/fl^ *Myo15‐cre^+/ki^* and *Clrn2* ^fl/fl^ *Myo15‐cre^+/ki^* mice and in about 50% of IHCs in *Clrn1* ^fl/fl^ *Clrn2* ^fl/fl^ *Myo15‐cre^+/ki^* mice; however, they were smaller in the absence of clarin‐1 (Figure 3B,C). Additionally, the number of BK clusters per IHC was significantly reduced in *Clrn1* ^fl/fl^ *Myo15‐cre^+/ki^*, *Clrn2* ^fl/fl^ *Myo15‐cre* ^+/ki^, and *Clrn1* ^fl/fl^ *Clrn2* ^fl/fl^ *Myo15‐cre* ^+/ki^ mice as compared to wild‐type mice (Figure 3D). Given the less severe phenotype found in these mice compared to clarin‐knockout mice, we measured BK channel currents (fast IK) in IHCs at P18‐24 (Figure 3E–G). BK currents were markedly decreased or absent in *Clrn1* ^fl/fl^ *Myo15‐cre* ^+/ki^ and *Clrn1* ^fl/fl^ *Clrn2* ^fl/fl^ *Myo15‐cre* ^+/ki^ mice and significantly reduced in *Clrn2* ^fl/fl^ *Myo15‐cre* ^+/ki^ mice (Figure 3E–G). Additionally, hair cell‐specific clarin knockout mice had significantly depolarized membrane potential, indicating chronic physiologic stress (Figure 3H). Overall, these alterations in BK channel function and expression mimic the alterations seen in the embryonic and constitutive knockout of clarin‐1 and clarin‐2 who largely lack MET currents.

### 2.2 Clarin‐1 and Clarin‐2 Are Required for Synaptic Organization and Function

**FIGURE 4:**
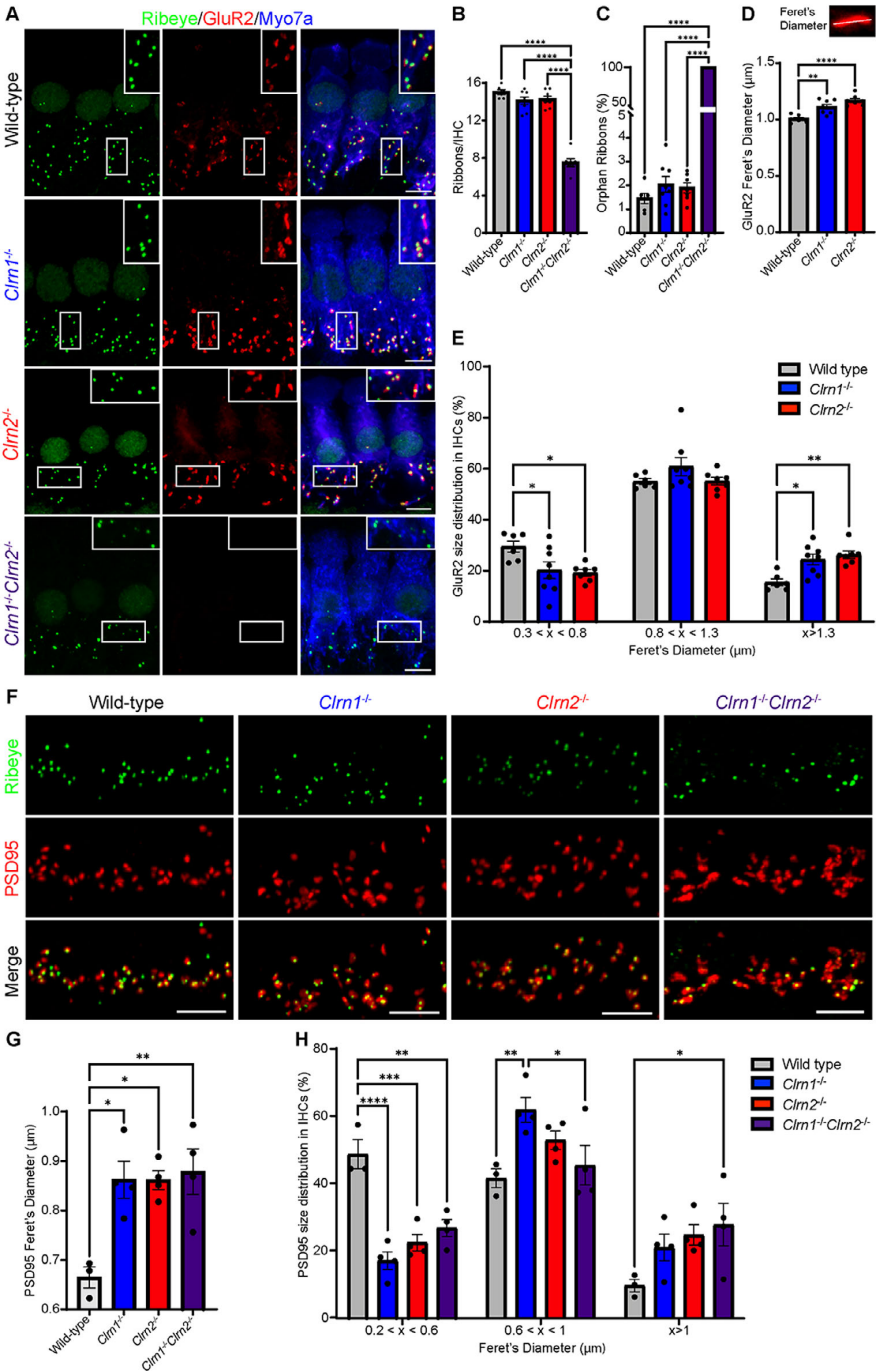
Clarin‐1 and clarin‐2 are required for inner hair cell synaptic organization. (A) Ribeye (green), GluR2 (red), and Myo7a (blue) immunostaining in IHCs of wild‐type, *Clrn1* ^−/−^, *Clrn2* ^−/−^, and *Clrn1* ^−/−^ *Clrn2* ^−/−^ mice at P21 at the middle turn of the cochlea, inset shows magnified synaptic active zone (scale bar = 5 µm). (B) Quantification of pre‐synaptic ribbons per IHC. (C) Quantification of percent of orphan ribbons (ribeye without GluR2 patch). (D) Quantification of overall GluR2 length via Feret's Diameter in synapses. (E) Distribution of GluR2 length categorized into small (0.3 µm < x < 0.8 µm), medium (0.8 µm < x < 1.3 µm), and large (x > 1.3 µm) glutamate receptors. Wild‐type *n* = 6, *Clrn1* ^−/−^ *n* = 8, and *Clrn2* ^−/−^ *n* = 7, with minimum 30 hair cells analyzed per *n*; mean ± SEM. (F) Ribeye (green) and PSD95 (red) immunostaining in IHCs of wild‐type, *Clrn1* ^−/−^, *Clrn2* ^−/−^, and *Clrn1* ^−/−^ *Clrn2* ^−/−^ mice at P21 at the middle turn of the cochlea (scale bar = 5 µm). (G) Quantification of overall PSD length via Feret's Diameter (H) Distribution of PSD95 length categorized into small (0.2 µm < x < 0.6 µm), medium (0.6 µm < x < 1 µm), and large (x > 1 µm). Wild‐type *n* = 3, *Clrn1* ^−/−^, *Clrn2* ^−/−^, and *Clrn1* ^−/−^ *Clrn2* ^−/−^ *n* = 4, with minimum 30 hair cells analyzed per *n*; mean ± SEM. Data were analyzed using one‐way ANOVA with Tukey's multiple comparisons test (B‐D, G), and two‐way ANOVA with Tukey's multiple comparisons test (E and H).

Previous work on *Clrn1* ^−/−^ mice revealed reduced calcium‐mediated exocytosis at IHC synapses [16]. We next asked whether the clarins also display a functional redundancy in synaptic organization and function. We first quantified the number of pre‐synaptic ribbons (labelled with ribeye) in mature IHCs (Figure 4A). *Clrn1* ^−/−^ *Clrn2* ^−/−^ mice had half the number of ribbons relative to wild‐type and single clarin knockout mice (Figure 4B). We next assessed the IHC synapses of clarin‐mutant mice (Figure 4A,C). Synapses were counted as the colocalization between pre‐synaptic ribbons (labelled with ribeye) and the post‐synaptic glutamate receptor 2 (labelled with GluR2), with orphan ribbons counted as pre‐synaptic ribbons lacking a post‐synaptic glutamate receptor. Strikingly, no GluR2 signal was detected in *Clrn1* ^−/−^ *Clrn2* ^−/−^ mice, therefore no true synapses were found, and yet there were no changes in the number of synapses or orphan ribbons in *Clrn1^−/^* ^−^ or *Clrn2* ^−/−^ mice (Figure 4C). Interestingly, the morphology of the post‐synaptic glutamate receptor, GluR2, is altered in *Clrn1^−/^* ^−^ and *Clrn2* ^−/−^ mice. Overall, GluR2 patches in *Clrn1^−/^* ^−^ and *Clrn2* ^−/−^ mice are elongated, as measured by Feret's diameter (Figure 4D). However, we did not observe a uniform elongation of the glutamate receptor, but rather a shift in the distribution of glutamate patch size (Figure 4E). The size of post‐synaptic glutamate receptors is dependent on the subtype and localization of afferent innervation [34]. Therefore, we categorized GluR2 size into small, medium, and large bins based on the mean and distribution of GluR2 patches in the wild‐type mice. In *Clrn1^−/−^* and *Clrn2* ^−/−^ mice, we found a reduction of the small pool of glutamate receptors with a shift to the largest pool of receptors, with no changes in medium‐sized receptors (Figure 4E).

Given the lack of GluR2 expression in *Clrn1* ^−/−^ *Clrn2* ^−/−^ mice, we further analyzed the post‐synaptic density by assessing the expression of post‐synaptic scaffolding protein, PSD95, in mature IHCs of wild‐type and clarin‐deficient mice (Figure 4F). PSD95 was elongated in *Clrn1* ^−/−^ and *Clrn2* ^−/−^ mice, while a striking disorganization and elongation of PSD95 was found in *Clrn1* ^−/−^ *Clrn2* ^−/−^ mice compared to wild‐type mice (Figure 4F,G). Although PSD95 size is not known to correlate with afferent innervation, we subdivided the size of PSD95 patches into small, medium, and large based on the overall distribution, as average Feret's diameter did not accurately represent the distinct changes we found in clarin‐mutant mice. In *Clrn1* ^−/−^ mice, we found a significant reduction in the smallest PSD95 patches with a shift to medium‐sized, while *Clrn2* ^−/−^ mice had only a significant reduction in small PSD95 patches, with only *Clrn1* ^−/−^ *Clrn2* ^−/−^ mice having a significant increase in large PSD95 patches (Figure 4H). The enhanced disorganization and expansion of the post‐synaptic density in *Clrn1* ^−/−^ *Clrn2* ^−/−^ mice could partially explain the lack of GluR2 expression in these mice.

**FIGURE 5:**
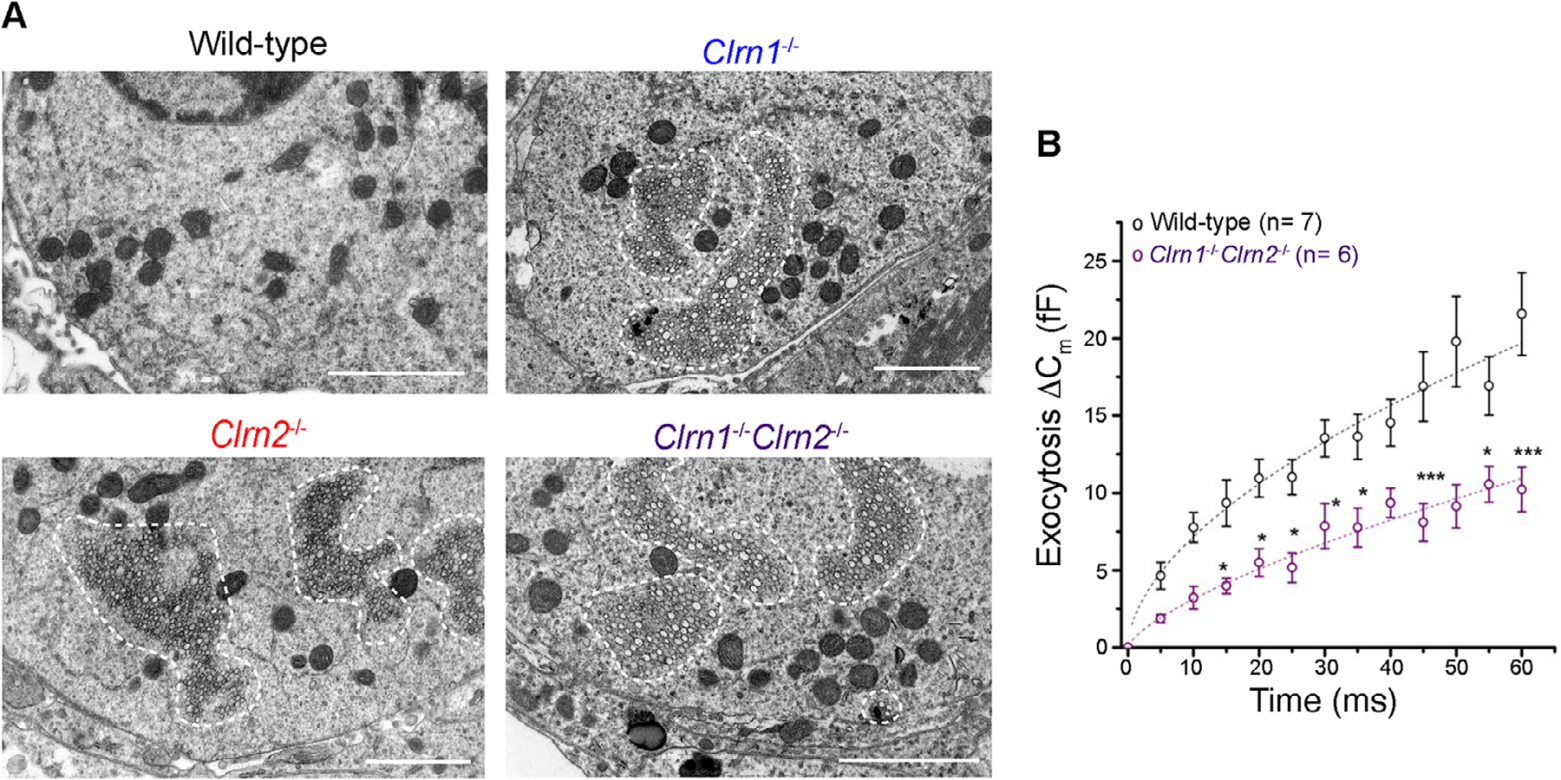
Clarin‐1 and clarin‐2 are required for proper inner hair cell synaptic function. (A) Transmission electron microscopy micrographs of the basolateral pole of IHCs of wild‐type, *Clrn1* ^−/−^, *Clrn2* ^−/−^, and *Clrn1* ^−/−^ *Clrn2* ^−/−^ mice at 1 month (scale bar = 2 µm). Dashed lines indicate infranuclear vesicle accumulations. (B) Comparative kinetics of exocytosis (depolarizing steps from −80 to −10 mV with increasing duration from 5 to 60 ms) in IHCs of Wild‐type (*n* = 7) and *Clrn1^−/−^Clrn2^−/−^* mice (*n* = 6) at P18‐P24. Data are presented as means ± SEM and were analyzed using two‐way ANOVA with Tukey's multiple comparisons test.

To dig deeper into the structural and functional ramifications of clarin‐1 and clarin‐2 deletion, we studied hair cells and primary auditory synapse structure at 1 month in wild‐type, *Clrn1* ^−/−^, *Clrn2* ^−/−^, and *Clrn1* ^−/−^ *Clrn2* ^−/−^ mice via transmission electron microscopy (TEM). Assessment of IHCs at this stage revealed no obvious defects in ribbon morphology, nor changes in mitochondrial morphology or integrity. However, a striking accumulation of infranuclear vesicles was found only in the cytoplasm of IHCs and at the active zone of all clarin‐mutant mice (Figure 5A). These vesicles are likely the consequence of impaired exocytosis and/or altered endocytosis and vesicle turnover in the absence of clarin‐1 and/or clarin‐2. Previous studies found reduced calcium‐mediated exocytosis in *Clrn1* ^−/−^ mice [16] and reduced ready release pool (RRP) exocytosis in *Clrn2* ^−/−^ mice [13]; therefore, we assessed exocytotic function in the absence of both clarin‐1 and clarin‐2. The kinetics of exocytosis in IHCs of *Clrn1* ^−/−^ *Clrn2* ^−/−^ mice were significantly reduced as compared to wild‐type mice (Figure 5B). Accordingly, a large portion of dysregulated genes in clarin‐deficient mice were implicated in synaptic function and organization in the transcriptomic analysis at P21 (Figure S5). Genes specifically implicated in glutamatergic synapse formation and function were predominantly down‐regulated in clarin‐deficient mice (Figure S5). Interestingly, we found significant reductions in the expressions of *Gria2*, encoding GluR2, *Grip2*, encoding a glutamate receptor interacting protein, and *Cacng2*, encoding stargazin, only in *Clrn1* ^−/−^ *Clrn2* ^−/−^ mice. Furthermore, there is an almost equal up‐ and down‐regulation of genes implicated in synaptic organization, bolstering the importance of clarin‐1 and clarin‐2 in the synaptic active zone (Figure S5). Therefore, we conclude that the lack of GluR2 expression in *Clrn1* ^−/−^ *Clrn2* ^−/−^ mice is a combination of its down‐regulation, a lack of structural support and organization in the post‐synaptic density, as well as altered functional aggregation of the receptor mediated by clarin‐1 and clarin‐2 in conjunction with known associating proteins.

**FIGURE 6:**
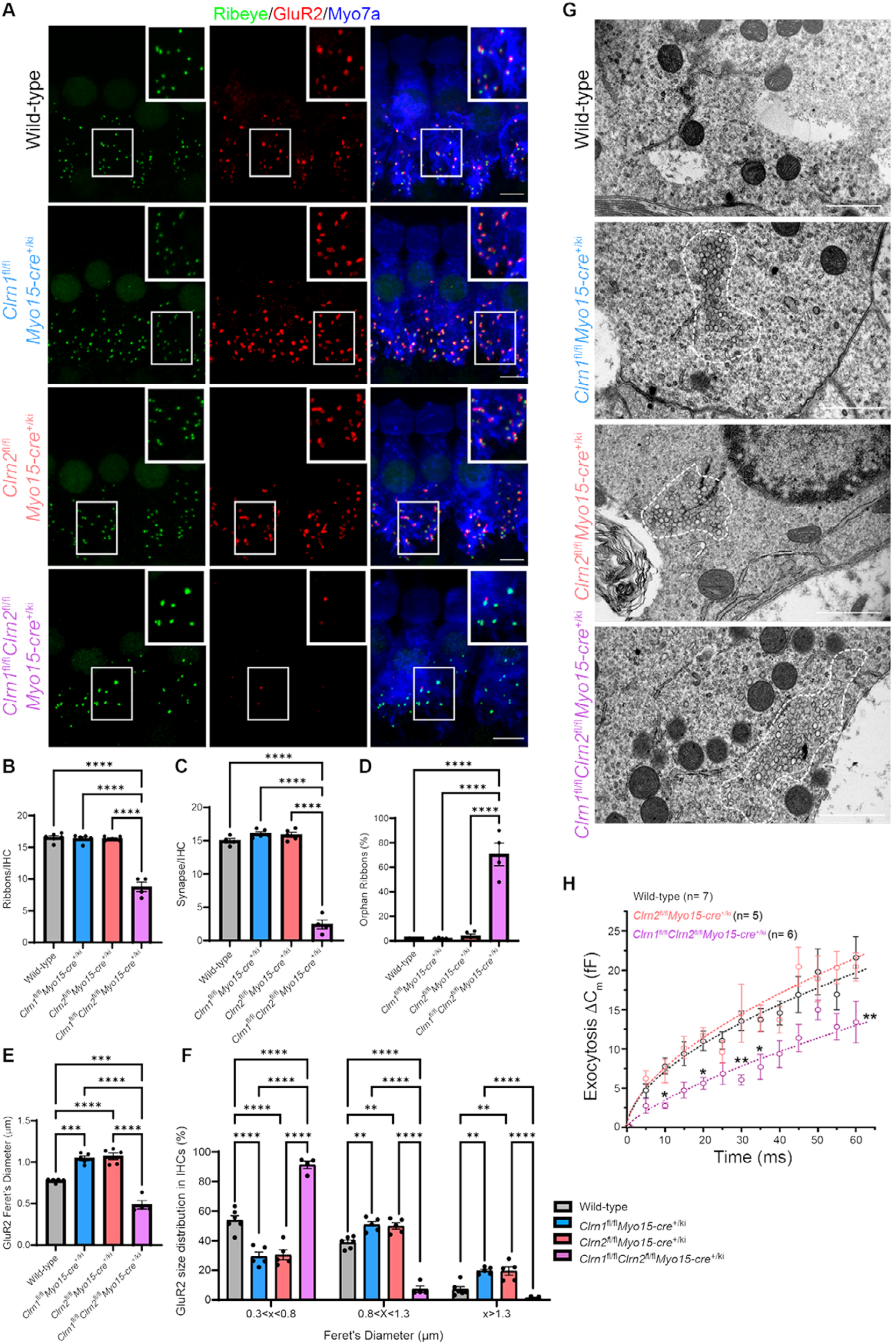
Clarin‐1 and clarin‐2 expression in hair cells is required for synaptic organization and function. (A) Ribeye (green), GluR2 (red), and Myo7a (blue) immunostaining in IHCs of wild‐type, *Clrn1* ^fl/fl^ *Myo15‐cre* ^+/ki^, *Clrn2* ^fl/fl^ *Myo15‐cre* ^+/ki^, and *Clrn1* ^fl/fl^ *Clrn2* ^fl/fl^ *Myo15‐cre* ^+/ki^ mice at P21 at the middle turn of the cochlea, inset shows magnified synaptic active zone (scale bar = 5 µm). (B) Quantification of pre‐synaptic ribbons per IHC. (C) Quantification of synapses (colocalization between ribeye and GluR2) (D) Quantification of percent of orphan ribbons (ribeye without GluR2 patch). (E) Quantification of overall GluR2 length via Feret's Diameter in synapses of P21. (F) Distribution of GluR2 length categorized into small (0.3 µm < x < 0.8 µm), medium (0.8 µm < x < 1.3 µm), and large (x > 1.3 µm) glutamate receptors. Wild‐type *n* = 6, *Clrn1* ^fl/fl^ *Myo15‐cre* ^+/ki^ and *Clrn2* ^fl/fl^ *Myo15‐cre* ^+/ki^ *n* = 5, *Clrn1* ^fl/fl^ *Clrn2* ^fl/fl^ *Myo15‐cre* ^+/ki^ *n* = 4, with minimum 30 hair cells analyzed per *n*; mean ± SEM. (G) Transmission electron microscopy micrographs of the basolateral pole of IHCs of wild‐type, *Clrn1* ^fl/fl^ *Myo15‐cre* ^+/ki^, *Clrn2* ^fl/fl^ *Myo15‐cre* ^+/ki^, and *Clrn1* ^fl/fl^ *Clrn2* ^fl/fl^ *Myo15‐cre* ^+/ki^ mice at 1 month (scale bar = 2 µm). Dashed lines indicate infranuclear vesicle accumulations. (H) Comparative kinetics of exocytosis (depolarizing steps from −80 to −10 mV with increasing duration from 5 to 60 ms) in IHCs of Wild‐type (*n* = 7), *Clrn2* ^fl/fl^ *Myo15‐cre* ^+/ki^ (*n* = 5), and *Clrn1* ^fl/fl^ *Clrn2* ^fl/fl^ *Myo15‐cre* ^+/ki^ (*n* = 6) mice at P18‐P24. Data are presented as mean ± SEM and were analyzed using one‐way ANOVA with Tukey's multiple comparisons test (B‐E) and two‐way ANOVA with Tukey's multiple comparisons test (F).

To determine whether these alterations are consequent to the roles clarin‐1 and clarin‐2 play specifically in hair cells, we assessed synaptic organization in mature IHCs from *Clrn1* ^fl/fl^ *Myo15‐cre* ^+/ki^, *Clrn2* ^fl/fl^ *Myo15‐cre* ^+/ki^, and *Clrn1* ^fl/fl^ *Clrn2* ^fl/fl^ *Myo15‐cre* ^+/ki^ mice (Figure 6). As expected, there were no alterations in ribbon or synaptic counts in the single hair cell‐conditional clarin knockout mice; however, *Clrn1* ^fl/fl^ *Clrn2* ^fl/fl^ *Myo15‐cre^+/ki^* mice had about half the number of presynaptic ribbons (Figure 6A–C). Unlike the *Clrn1* ^−/−^ *Clrn2* ^−/−^ mice, around 30% of IHCs of *Clrn1* ^fl/fl^ *Clrn2* ^fl/fl^ *Myo15‐cre* ^+/ki^ mice expressed GluR2 and had true synapses, while the majority of IHCs had only orphan ribbons (Figure 6C,D).

As in single total clarin knockout mice, we found an elongation of glutamate receptor, GluR2, in *Clrn1* ^fl/fl^ *Myo15‐cre* ^+/ki^ and *Clrn2* ^fl/fl^ *Myo15‐cre* ^+/ki^ mice with a reduction in the smallest patches and a shift toward medium and large GluR2 patches in these conditional knockout mice (Figure 6E,F). Interestingly, when GluR2 was expressed in *Clrn1* ^fl/fl^ *Clrn2* ^fl/fl^ *Myo15‐cre* ^+/ki^ mice, the patches were significantly smaller than those of wild‐type mice, with the majority of GluR2 patches falling in the smallest size range (Figure 6E,F). These alterations in GluR2 morphology found in hair cell‐specific clarin knockout mice indicate that clarin‐1 and clarin‐2 are required for the maintenance of GluR2 expression due to the synaptic disorganization in their absence and confirm that the synaptic alterations found in total clarin‐mutant mice are derived from the pre‐synaptic role(s) of clarin‐1 and clarin‐2 in hair cells. Furthermore, we again found this striking infranuclear vesicular accumulation in IHCs of all hair cell‐specific clarin conditional knockout mice at P30 (Figure 6G). Previous work on *Clrn1* ^fl/fl^ *Myo15‐cre* ^+/ki^ mice revealed a significant impairment in exocytosis [16]; therefore, we only assessed *Clrn2* ^fl/fl^ *Myo15‐cre* ^+/ki^ and *Clrn1* ^fl/fl^ *Clrn2* ^fl/fl^ *Myo15‐cre* ^+/ki^ mice at P18‐P24. Interestingly, *Clrn2* ^fl/fl^ *Myo15‐cre* ^+/ki^ mice had normal exocytosis kinetics, while exocytosis in *Clrn1* ^fl/fl^ *Clrn2* ^fl/fl^ *Myo15‐cre* ^+/ki^ mice was significantly reduced (Figure 6H).

### 2.3 Clarin‐1 and Clarin‐2 Are Required for Hair Cell and Primary Auditory Neuron Survival

**FIGURE 7:**
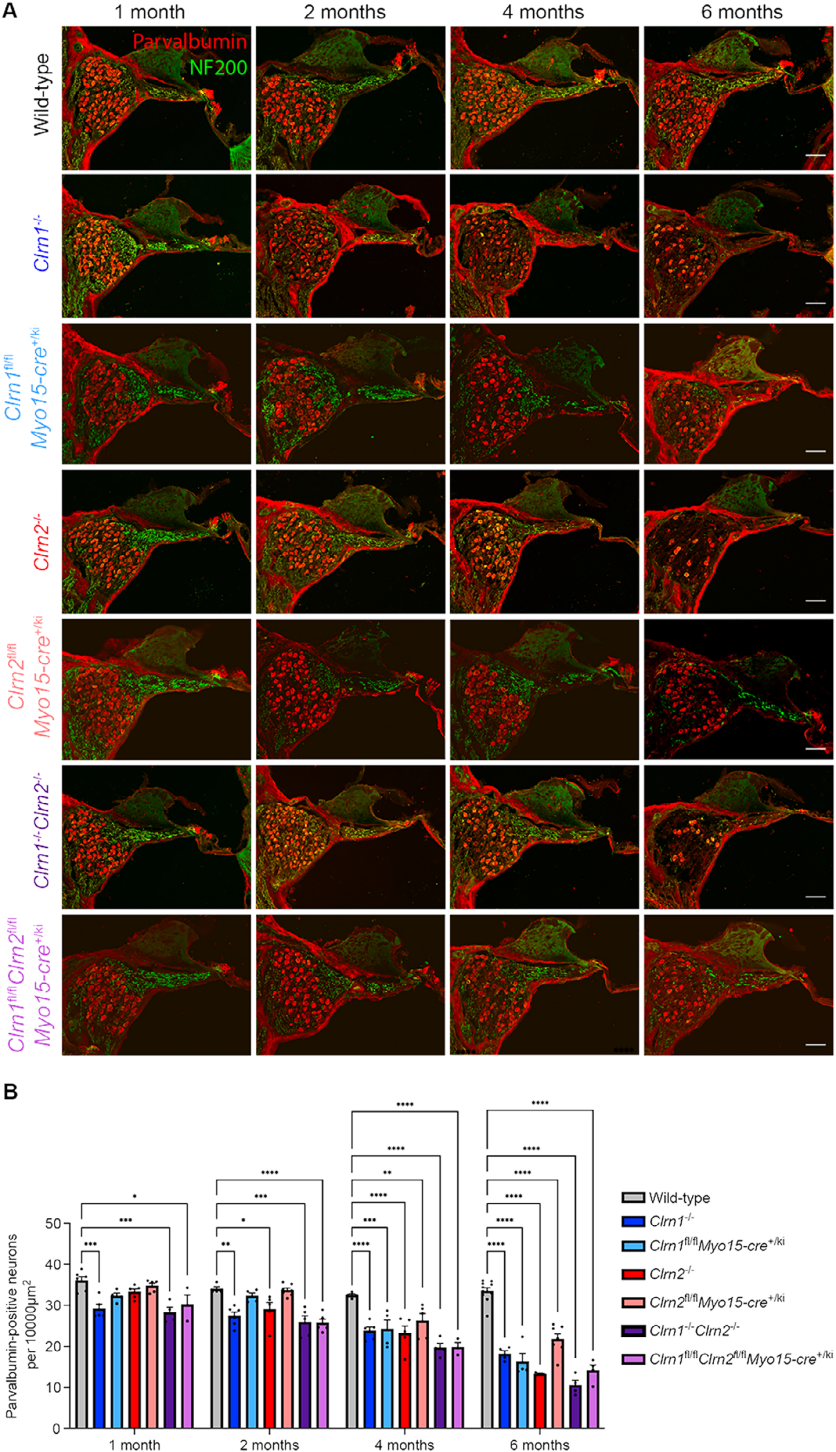
Clarin‐1 and clarin‐2 expression in hair cells is required for primary auditory neuron survival. (A) Parvalbumin (red) and Neurofilament 200 (green) immunostaining of primary auditory neurons and hair cells in wild‐type, *Clrn1* ^−/−^, *Clrn1* ^fl/fl^ *Myo15‐cre* ^+/ki^, *Clrn2* ^−/−^, *Clrn2* ^fl/fl^ *Myo15‐cre* ^+/k^ *^i^*, *Clrn1* ^−/−^ *Clrn2* ^−/−^, and *Clrn1* ^fl/fl^ *Clrn2* ^fl/fl^ *Myo15‐cre* ^+/ki^ mice at 1 month (first column), 2 months (second column), 4 months (third column), and 6 months (fourth column) at the middle turn of the cochlea (scale bar = 50 µm). (B) Quantification of parvalbumin‐positive neurons per 10 000 µm^2^ (normalized to the area of Rosenthal canal) at 1, 2, 4, and 6 months. All genotypes and ages *n *≥ 3; data are mean ± SEM; analyzed by two‐way ANOVA with Tukey's multiple comparisons test.

We hypothesized that primary auditory neuron degeneration would occur in the absence of clarin‐1 and/or clarin‐2 consequent to clarin‐related hair cell dysfunction. We first assessed clarin‐1 and clarin‐2 expression in the primary auditory neurons using RNAscope. We found clarin‐1 and clarin‐2 to be expressed in the majority of primary auditory neurons, 99% and 97%, respectively (Figure S6). Next, primary auditory neurons and their afferent fibers in the middle turn of wild‐type and total and condition single and double clarin knockout mice at 1, 2, 4, and 6 months were assessed using parvalbumin and neurofilament heavy chain (NF200) (Figure 7A). At 1 month, there was a significant, but moderate loss (∼20%) of primary auditory neurons in *Clrn1* ^−/−^ and *Clrn1* ^−/−^ *Clrn2* ^−/−^ mice, while *Clrn2* ^−/−^ mice had a nonsignificant loss of ∼10% of primary neurons relative to wild‐type (Figure 7B). In contrast, in the hair‐specific knockout mice, at 1 month, only *Clrn1* ^fl/fl^ *Clrn2* ^fl/fl^ *Myo15‐cre^+/ki^* mice had significant losses relative to wild‐type, with ∼15%, with a nonsignificant average loss of ∼10% in *Clrn1* ^fl/fl^ *^l^Myo15‐cre* ^+/ki^ mice, but by 4 months of age, all hair cell‐specific knockout mice had significant losses (Figure 7B). *Clrn1* ^fl/fl^ *Myo15‐cre* ^+/ki^ and *Clrn1* ^fl/fl^ *Clrn2* ^fl/fl^ *Myo15‐cre* ^+/ki^ mice had significant primary auditory neuron losses, ∼25% and ∼40%, respectively, with losses in *Clrn2* ^fl/fl^ *Myo15‐cre* ^+/ki^ mice (∼20%) (Figure 7B). By 6 months of age *Clrn2* ^−/−^ and *Clrn1* ^−/−^ *Clrn2* ^−/−^ mice had severe loss (∼65%–70%) with nearly empty Rosenthal canals, while *Clrn1* ^−/−^ mice had sustained a moderate loss of ∼45% relative to age‐matched controls (Figure 7B). *Clrn1* ^−/−^ *Clrn2* ^−/−^ mice presented with an earlier onset and more severe degeneration of the sensory epithelium, which in some mice was completely collapsed by 2 months of age, and in all mice by 4 months of age, while *Clrn1^−/^* ^−^ and *Clrn2^−/−^* mice had sensory epithelial collapses at 6 and 4 months respectively (Figure 7A). At 6 months of age, neuronal degeneration progressed steadily to ∼50% loss in *Clrn1* ^fl/fl^ *Myo15‐cre* ^+/ki^ mice, ∼60% loss in *Clrn1* ^fl/fl^ *Clrn2* ^fl/fl^ *Myo15‐cre* ^+/ki^ mice, and ∼35% loss in *Clrn2* ^fl/fl^ *Myo15‐cre* ^+/ki^ mice (Figure 7B). Interestingly, the sensory epithelium completely collapsed in *Clrn1* ^fl/fl^ *Myo15‐cre* ^+/ki^ and *Clrn1* ^fl/fl^ *Clrn2* ^fl/fl^ *Myo15‐cre* ^+/ki^ mice by 4 months of age, whereas degenerative changes started occurring at 6 months in *Clrn2* ^fl/fl^ *Myo15‐cre* ^+/ki^ (Figure 7).

Assessment of primary auditory neurons at 1 month in total and hair cell‐specific knockout of clarin‐1 and/or clarin‐2 by TEM revealed no neuronal swellings nor alterations in myelination in clarin‐mutant mice; however, structural mitochondrial anomalies were found in ∼40%–55% of neuronal mitochondria from all clarin‐mutant mice, a sign of mitochondrial degeneration (Figure S7). Mitochondrial swelling, with disrupted cristae, was found in all clarin‐mutant mice, as well as mitochondria lacking normal cristae, with internalized vesicles and or vacuoles (Figure S7). These vesicles do not appear to be mitochondrial‐derived vesicles, which normally present as vesicles budding from the outer mitochondrial membrane, used for intracellular signaling, but rather a pathological hallmark of mitochondrial dysfunction and degeneration. Alterations in the expression of genes implicated in mitochondrial function (oxidative phosphorylation and glucose metabolism) and survival (oxidative stress and pro‐aptotic cytochrome c release) were also detected in the transcriptomic analysis (Figure S8), indicating that these structural mitochondrial changes have functional outcomes. Overall, the transcriptional changes paired with the structural hallmarks of mitochondrial dysfunction found in clarin‐mutant mice provide evidence that early mitochondrial dysfunction is implicated in primary auditory neuron degeneration in the absence of clarin‐1 and/or clarin‐2.

### 2.4 Clarin‐1 and Clarin‐2 Expression in Primary Auditory Neurons Is Not Required for Primary Auditory Neuron Survival or Audition

As we hypothesized, primary auditory neuron degeneration is consequent to clarin‐mediated dysfunction in hair cells, we generated primary auditory neuron‐specific conditional clarin knockout mice by crossing *Clrn1* ^fl/fl^ and *Clrn2* ^fl/fl^ mice with *Bhlhb5‐cre* mice.

We first characterized the expression of the *Bhlhb5‐cre*, using the reporter line *Bhlhb5‐cre* ^+/ki^ *Rosa‐TdTomato* ^+/ki^ (Figure S9A). We found that TdTomato expression was present only in primary auditory neurons and their afferent fibers, and expression was stable and in all type I primary auditory neurons in the middle and basal turns of the cochlea from P21 to 6 months of age (Figure S9B). Overall, this indicated that clarin‐1 and clarin‐2 deletion in *Bhlhb5‐cre* mice is specific and durable, with the majority of all type I primary auditory neurons lacking clarin‐1 or clarin‐2.

**FIGURE 8:**
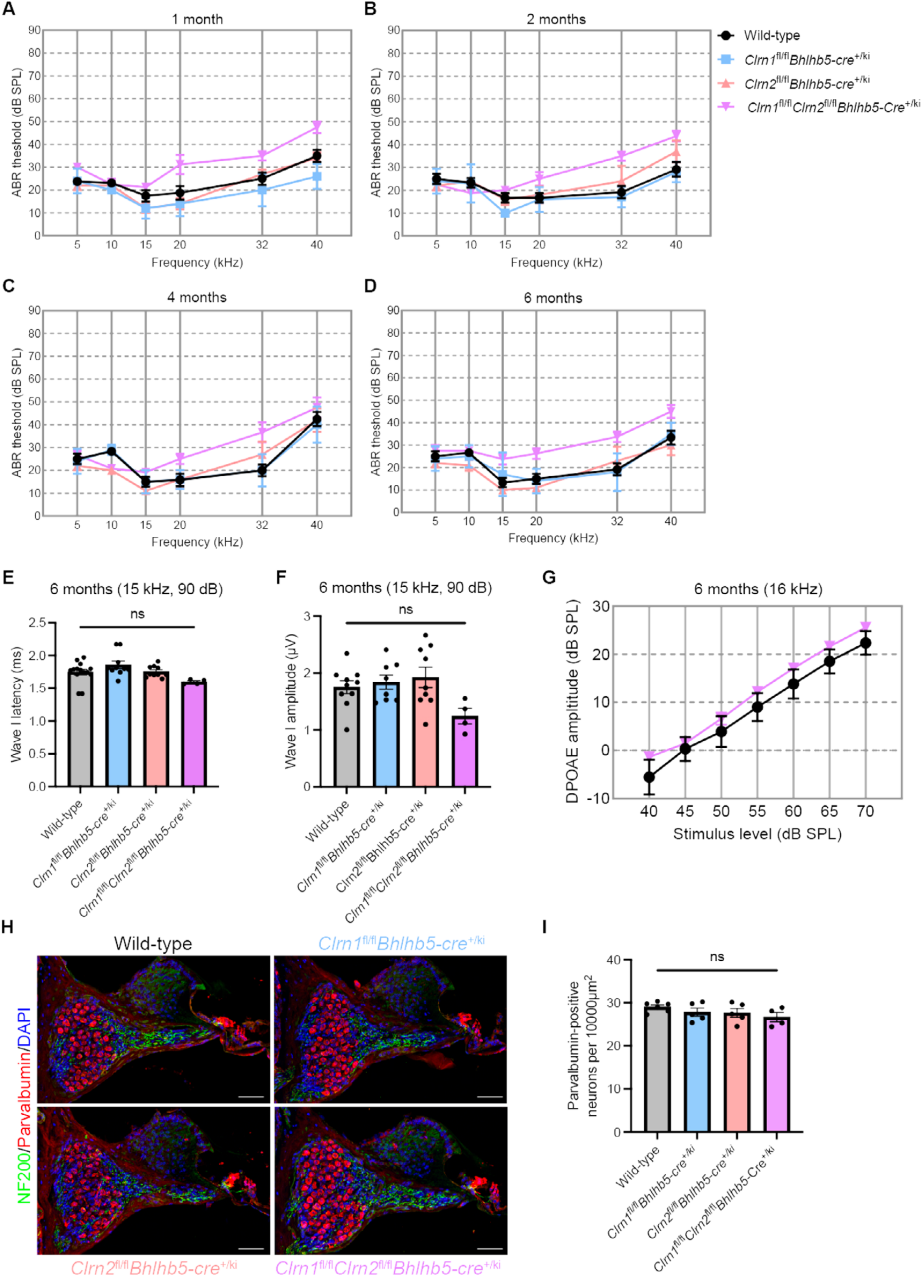
Clarin‐1 and clarin‐2 expression in primary auditory neurons not required for hearing or primary auditory neuron survival. ABR thresholds in wild‐type (black trace), *Clrn1* ^fl/fl^ *Bhlhb5‐cre* ^+/ki^ (blue trace), *Clrn2* ^fl/fl^ *Bhlhb5‐cre* ^+/ki^ (red trace), and *Clrn1* ^fl/fl^ *Clrn2* ^fl/fl^ *Bhlhb5‐cre* ^+/ki^ (purple trace) mice at (A) 1 month, (B) 2 months, (C) 4 months, and (D) 6 months of age. Wave I (E) amplitude and (F) latency at 15 kHz, measured at 90 dB SPL at 6 months. Wild‐type *n *≥ 6, *Clrn1* ^fl/fl^ *Bhlhb5‐cre* ^+/ki^ *n *≥ 5, *Clrn2* ^fl/fl^ *Bhlhb5‐cre* ^+/ki^ *n *≥ 5, and *Clrn1* ^fl/fl^ *Clrn2* ^fl/fl^ *Bhlhb5‐cre* ^+/ki^ *n* = 4 at all time points. (G) DPOAE amplitudes of wild‐type (black trace) and *Clrn1* ^fl/fl^ *Clrn2* ^fl/fl^ *Bhlhb5‐cre* ^+/ki^ (purple trace) mice at 6 months for 16 kHz. (H) Parvalbumin (red), Neurofilament 200 (green), and DAPI (blue) immunostaining of primary auditory neurons and hair cells of wild‐type, *Clrn1* ^fl/fl^ *Bhlhb5‐cre* ^+/ki^, *Clrn2* ^fl/fl^ *Bhlhb5‐cre* ^+/ki^, *Clrn1* ^fl/fl^ *Clrn2* ^fl/fl^ *Bhlhb5‐cre* ^+/ki^ mice at 6 months in the basal turn of the cochlea (scale bar = 50 µm). (I) Quantification of parvalbumin‐positive neurons per 10000µm^2^ (normalized to area of Rosenthal canal). Wild‐type *n* = 6, *Clrn1* ^fl/fl^ *Bhlhb5‐cre* ^+/ki^ and *Clrn2* ^fl/fl^ *Bhlhb5‐cre* ^+/ki^ *n* = 5, *Clrn1* ^fl/fl^ *Clrn2* ^fl/fl^ *Bhlhb5‐cre* ^+/ki^ *n* = 4. Data are presented as mean ± SEM and were analyzed using one‐way ANOVA with Tukey's multiple comparisons test.

To determine the contribution of clain‐1 and clarin‐2 in primary auditory neurons to audition, we recorded ABRs in *Clrn1* ^fl/fl^ *Bhlhb5‐cre* ^+/ki^ mice, *Clrn2* ^fl/fl^ *Bhlhb5‐cre* ^+/ki^, and *Clrn1* ^fl/fl^ *Clrn2* ^fl/fl^ *Bhlhb5‐cre* ^+/ki^ mice over time. Auditory thresholds were the same as those of age‐matched controls from 1 to 6 months of age (Figure 8A–D). Furthermore, in aged mice, there were no significant changes in wave I amplitude or latency (Figure 8E,F), indicating no alteration in synaptic transmission or auditory neuropathology associated with the primary auditory neuron‐specific deletion of clarin‐1 and/or clarin‐2. Furthermore, DPOAE amplitudes in *Clrn1* ^fl/fl^ *Clrn2* ^fl/fl^ *Bhlhb5‐cre* ^+/ki^ mice were not significantly different from those of wild‐type mice 6 months of age (Figure 8G), indicating normal OHC function.

Finally, we assessed the survival of primary auditory neurons, their afferent fibers, and the sensory epithelium at 6 months of age in *Clrn1* ^fl/fl^ *Bhlhb5‐cre* ^+/ki^, *Clrn2* ^fl/fl^ *Bhlhb5‐cre* ^+/ki^, and *Clrn1* ^fl/fl^ *Clrn2* ^fl/fl^ *Bhlhb5‐cre* ^+/ki^ mice (Figure 8H). We observed no degeneration of primary auditory neurons in the basal turns of the cochlea (Figure 8I), and there were no signs of hair cell or supporting cell degeneration in the sensory epithelium. Additionally, we assessed the neuronal mitochondria via TEM of *Clrn1* ^fl/fl^ *Clrn2* ^fl/fl^ *Bhlhb5‐cre* ^+/ki^ mice at 1 month of age; as expected, the mitochondria did not show any signs of damage or degeneration (Figure S7). Therefore, we conclude that the primary role(s) of clarin‐1 and clarin‐2 are in hair cells, and their expression in the primary auditory neurons is not required for audition.

## 3 Discussion

Usher Syndrome is the number one cause of deaf‐blindness; however, there exist few viable treatment options due to our limited understanding of the roles that the causative genes play in the auditory system. Usher Syndrome type III patients, with mutations in clarin‐1, suffer from extremely variable auditory phenotypes. We hypothesized that this phenotypic variability is due, at least in part, to the interplay between clarin‐1 and its paralog, clarin‐2, which has recently been linked to non‐syndromic hearing loss [11, 13]. Here, we provide a comprehensive analysis of clarin‐1 and clarin‐2 function in the inner ear using genetic mouse models—including total knockouts, cell type‐specific deletions, and double mutants. Our findings reveal a previously unrecognized compensatory interaction between the two proteins, whose co‐expression is essential for mechanoelectrical transduction, synaptic transmission, and primary auditory neuron survival. These results offer critical mechanistic insight into the progression and heterogeneity of hearing loss and carry direct clinical relevance for cochlear implantation, as well as genetic diagnosis and prognosis in hereditary deafness.

One of the most striking findings of this study is the complete loss of mechanoelectrical transduction (MET) currents in *Clrn1* ^−/−^ *Clrn2* ^−/−^ mice, accompanied by a profound auditory phenotype. As previously reported, we found that loss of either clarin‐1 or clarin‐2 alone only leads to a reduction in MET currents [10, 16, 23], demonstrating a compensatory role between the clarins in MET function. This identifies clarins as previously unrecognized putative accessory components of the MET complex. Similar functional redundancy exists for core MET players, such as TMC1 and its paralog TMC2, while *Tmc1* ^−/−^ mice retain MET currents before P7 due to compensatory TMC2 expression [35, 36], double *Tmc1* ^−/−^ *Tmc2* ^−/−^ mice lack MET currents entirely [37, 38].

Further support for the role of clarins in MET function is the compensatory upregulation of genes directly implicated in the MET complex, *Tmc1* and protocadherin‐15 (*Pcdh15*). TMC1 is thought to be the pore‐forming MET channel, while PCDH15 forms the lower tip‐link crucial to MET channel opening [39, 40, 41, 42, 43]. Together, TMC1 and PCDH15, with TMIE, LHFPL5, and CIB2 make up the known components of the MET complex [24, 25, 26, 44, 45, 46, 47, 48]. To position clarins within this framework, we used bimolecular fluorescence complementation (BiFC), a membrane‐preserving approach well suited for probing interactions among hydrophobic MET proteins [49]. These assays revealed that clarin‐1 engages a broad set of MET‐associated partners, including TMC1, TMIE, LHFPL5, PCDH15, CDH23, and harmonin, whereas clarin‐2 displays a more restricted interaction profile, overlapping primarily with clarin‐1, TMIE, harmonin, and LHFPL5. This partial overlap in interaction partners provides a molecular basis for functional compensation in single knockouts and the complete collapse of MET in *Clrn1* ^−/−^ *Clrn2* ^−/−^ mice. Of equal importance in MET function is the stability and organization of stereocilia in the hair bundle. Our *Clrn1* ^−/−^ *Clrn2* ^−/−^ mice display severe bundle fragmentation beyond that seen in single knockouts, pointing to additive roles in bundle stability. Loss of individual stereocilia may result from disrupted calcium influx due to reduced MET activity, destabilizing the actin core. The observed upregulation of *Diaph1* and *Espnl* in both RNAseq and qPCR datasets suggests compensatory responses aimed at stabilizing actin cores in the absence of clarin proteins, though excessive *Diaph1* activity may paradoxically promote bundle disruption [50].

Together, these data position clarin‐1 and clarin‐2 as essential structural and functional components of the MET complex and hair bundle architecture, working cooperatively to preserve transduction capacity (see model, Figure 1I).

Our findings also reveal profound disruption of ionic homeostasis in clarin‐deficient hair cells, which is intrinsically linked to mechanoelectrical transduction. PMCA2, the key calcium extrusion pump, was abnormally expressed in IHC stereocilia across all P21 clarin‐mutant mice—consistent with an immature, pre‐hearing state [51]. Upregulation of *P2ry1*, a purinergic receptor involved in pre‐hearing spontaneous activity [17], further supports delayed maturation. Overall, the overactive calcium extrusion in this context may deplete stereocilia calcium to levels that destabilize actin filaments, leading to bundle degeneration [52].

Potassium signaling was equally affected. BK channels, which facilitate rapid repolarization of IHCs and are essential for adult synaptic precision [31, 33, 53, 54], were reduced in single clarin mutants and abolished in *Clrn1* ^−/−^ *Clrn2* ^−/−^ mice. The hair cell‐specific conditional knockouts showed that both the reduction in BK currents and altered voltage activation profiles arise from hair cell‐autonomous loss of clarins. RNA‐seq analysis further revealed that *Ryr1*, which modulates BK activity and co‐localizes with BK channels [53, 54], was significantly upregulated in all clarin mutants, possibly as a compensatory attempt to activate BK currents. Conversely, *Lrrc52*, the γ subunit critical for BK channel activation at physiological voltages (−60 to −90 mV), was significantly downregulated. Loss of *Lrrc52* shifts BK activation by +200 mV and disrupts channel clustering [55], consistent with reduced or absent BK channel clusters observed in clarin‐deficient mice.

Overall, clarin‐1 and clarin‐2 play a key role in the maintenance of calcium and potassium homeostasis via the functional changes induced in potassium and calcium channels in their absence, primarily due to clarin‐mediated MET dysfunction.

Notably, we observed a dramatic loss of synaptic integrity in the absence of both clarin‐1 and clarin‐2, with the two proteins acting at both pre‐ and post‐synaptic levels through MET‐dependent and structural mechanisms. Our data support a model in which presynaptic and postsynaptic alterations arise through partially distinct mechanisms. In single clarin knockouts, presynaptic ribbon counts were unchanged; however, in *Clrn1* ^−/−^ *Clrn2* ^−/−^ and *Clrn1* ^fl/fl^ *Clrn2* ^fl/fl^ *Myo15‐cre* ^+/ki^ mice, ribbon numbers were reduced by ∼50%, which is likely due to the complete lack of MET in these mice. Indeed, when MET is greatly reduced or abolished in *TMC1*, *TMC1*/*TMC2*, or *TMIE* knockout mice [37, 38, 56], there are around half the number of presynaptic ribbons in mature IHCs relative to wild‐type [56], indicating that MET function is required for normal ribbon development and maintenance. Therefore, we propose that the reduction in pre‐synaptic ribbons found in *Clrn1* ^−/−^ *Clrn2* ^−/−^ mice, is a secondary consequence of abolished MET currents. Consistent with this interpretation, transcriptomic analysis did not reveal significant downregulation of Ctbp2, suggesting that Ribeye loss reflects post‐transcriptional destabilization of synaptic ribbons rather than reduced gene expression. While ribbon counts remained normal in single mutants, significant changes in GluR2 morphology were observed, with a shift from small to large patches—features that have been associated with altered afferent subtype distribution and innervation patterning [34].

Post‐synaptically, both single mutants showed elongation of glutamate receptor GluR2 and scaffolding protein PSD95, indicating reduced post‐synaptic density (PSD) cohesion. In contrast to the presynaptic phenotype, these postsynaptic alterations are unlikely to be explained solely by reduced MET activity, as similar changes have not been reported in other MET‐deficient mouse models. These changes resemble AMPA receptor mislocalization seen when stargazin–PSD95 interactions are disrupted in the central nervous system [57, 58, 59], pointing to a similar role for clarins in cochlear synapses. In double knockouts, synaptic disorganization was more severe: GluR2 was undetectable, PSD95 appeared disorganized and enlarged, and *Cacng5* (stargazin) was significantly downregulated. Given the known roles of PSD95 and stargazin in AMPA receptor trafficking, these findings suggest that the clarins act in tandem with this pathway to stabilize glutamate receptor complexes at the synapse. Therefore, we propose that clarin‐1 and clarin‐2 are directly mediating the changes in post‐synaptic architecture and stability.

Transcriptomic profiling confirmed downregulation of key glutamatergic signaling genes, including *Gria2* (GluR2), *Grip2* (a glutamate receptor‐interacting protein), and *Cacng2* (stargazin), all essential for receptor clustering and function [57, 58, 59]. Given their four‐transmembrane structure, clarins may function similarly to stargazin in stabilizing AMPA receptors [14].

Functional assays in all *Clrn1*‐deficient mice confirmed reduced exocytosis and vesicle release defects, with TEM showing vesicle accumulation near the active zone of clarin‐deficient IHCs, consistent with impaired vesicle release and turnover. Dysregulation of caveolae‐mediated endocytosis pathways suggests that impaired recycling may contribute to vesicle buildup and cytotoxicity—exacerbated by calcium imbalance. Taken together, these data support a model in which clarin‐1 and clarin‐2 influence synaptic integrity through both indirect, MET‐dependent effects on presynaptic maintenance and direct, MET‐independent roles in organizing postsynaptic receptor complexes. These findings reinforce a model in which the two clarin proteins are central to pre‐ and post‐synaptic architecture and signaling via their roles in MET function and their structural role in the synapse.

Primary auditory neurons' survival is critically dependent on clarin‐1 and clarin‐2 function within hair cells. Constitutive and hair cell‐specific knockouts of *Clrn1* and *Clrn2* led to progressive primary auditory neuron degeneration, closely matching the severity and timing of sensory epithelial collapse. By six months, all clarin knockout mice had upward of 40% primary auditory neuron degeneration, underscoring the essential role of hair cell‐derived clarins in maintaining afferent neuronal integrity. Although both clarins are expressed in nearly all primary auditory neurons, neuron‐specific deletion using *Bhlhb5‐Cre* had no effect on auditory thresholds, synaptic transmission, or primary auditory neuron survival, even at later stages in single or double knockout mice. This demonstrates that clarin expression is not intrinsically required within primary auditory neurons.

Ultrastructural TEM analysis revealed mitochondrial pathology—including swelling and vacuolization—in primary auditory neurons of total and hair cell‐specific clarin‐deficient mice, and RNA‐seq confirmed upregulation of pro‐apoptotic *BCL2* family genes (*Bcl2l11, Hrk, Bik*), oxidative stress markers, and regulators of mitochondrial depolarization. Given that there were no alterations in hair cell mitochondria, these changes point to a secondary effect of mitochondrial‐driven apoptosis in the neurons rather than a clarin‐mediated mitochondrial dysfunction resulting in primary auditory neuron degeneration.

These mechanistic findings have two direct clinical implications for Usher syndrome type III patients. First, functional redundancy between clarin‐1 and clarin‐2 has broad implications for understanding phenotypic variability in patients with monogenic forms of deafness. In humans, mutations in *CLRN1* can lead to a spectrum of auditory phenotypes ranging from mild progressive loss to profound congenital deafness. Our data reveal that *CLRN1* mutation‐related symptoms may, in part, be impacted by the absence of functional *CLRN2* alleles. This suggests that *CLRN2* variants may worsen *CLRN1*‐associated deafness, with variable penetrance among individuals, due to disruptions in the MET complex's structural or functional integrity. As such, comprehensive genetic screening for individuals with *CLRN1* mutations should include *CLRN2*, particularly in cases with unexpectedly severe or variable presentations. Second, early intervention with cochlear implants could be life‐altering, particularly for patients with mutations in both clarin‐1 and clarin‐2. Given that neuronal demise is consequent to clarin‐mediated hair cell dysfunction, if detected early enough, these patients would be excellent candidates for cochlear implants (CI). Cochlear implants bypass dysfunctional hair cells to directly stimulate primary auditory neurons. Our findings suggest that primary auditory neurons remain structurally intact in the absence of clarins, provided they continue to receive input—either through natural hair cell function or electrical stimulation. This underscores the importance of early implantation in USH3 deafness to preserve auditory nerve integrity and maximize CI outcomes. Timely intervention may prevent the secondary neurodegeneration observed in clarin‐deficient models, offering a promising therapeutic window for effective hearing restoration. Indeed, a recent study following up on the long‐term implications of cochlear implantation in USH3 patients supports this hypothesis [60]. Recent successes of clarin‐based viral gene supplementation strategies [13, 16, 61, 62], together with emerging gene therapy approaches for hereditary deafness in humans, including recent AAV‐OTOF trials demonstrating robust auditory improvement [63, 64], underscore the strong translational potential of clarin‐targeted therapeutic strategies.

In conclusion, these findings redefine how we understand and approach “monogenic” deafness. Rather than just an isolated defect in *CLRN1*, Usher Syndrome type III hearing loss phenotypes may emerge from a network of interacting genes and compensatory mechanisms. Integrating this systems‐level perspective into diagnostic pipelines [65] and therapeutic design promises not only more accurate risk prediction and patient stratification but also more effective, personalized treatment strategies.

## 4 Methods

### 4.1 Animal Handling

Animals were housed in the Institut Pasteur animal facilities, which are accredited by the French Ministry of Agriculture for experimentation on live mice (accreditation 75‐15‐01, issued on September sixth, 2013) in application of the French and European regulations on the care and protection of laboratory animals (EC Directive 2010/63, French Law 2013–118, February sixth, 2013). Animal testing was carried out with the approval of the Institut Pasteur ethics committee (Institut Pasteur‐DAP180039 and DAP240030).

### 4.2 Generation of Clarin‐1 and Clarin‐2 Mutant Mice

To obtain clarin‐1 total knockout mice, *Clrn1^ex4^* ^fl/fl^ mice (LoxP sites on both sides of *Clrn1* exon 4), after deletion of the *neo* cassette, were crossed with *PGK‐cre^+/−^* mice resulting in an embryonic and constitutive deletion of clarin‐1 (*Clrn1^ex4−/−^* mice) [16], hereafter referred to as *Clrn1^−/−^* mice. Hair cell‐specific conditional clarin‐1 knockout mice were obtained by crossing *Clrn1^ex4^* ^fl/fl^ mice with *Myo15‐cre* ^+/ki^ mice [66], resulting in a post‐natal deletion of clarin‐1 restricted to inner and outer hair cells [16]. Primary auditory neuron‐specific clarin‐1 knockout mice were obtained by crossing *Clrn1^ex4^* ^fl/fl^ mice with *Bhlhb5‐cre* ^+/ki^ mice, resulting in a post‐natal deletion of clarin‐1 restricted to the primary auditory neurons [67] and non‐auditory neurons of the central nervous system [68, 69].

*Clrn2* ^clarinet/clarinet^ mice, containing a tryptophan‐to‐stop (p.Trp4*) nonsense mutation resulting in the embryonic and constitutive deletion of clarin‐2, were used as total clarin‐2 knockout mice, hereafter referred to as *Clrn2* ^−/−^ mice [10]. The C57BL/6NTac‐Clrn2^em2H^/H (Clrn2^ex2fl/fl^; LoxP sites on both sides of *Clrn2* exon 2) mouse line was generated on a C57BL/6N background by MRC Harwell Institute using CRISPR‐Cas9 genome editing and long single‐stranded DNA donors [70, 71] (Table S4). Briefly, microinjection buffer (MIB; 10 mm Tris–HCl, 0.1 mm EDTA, 100 mm NaCl, pH 7.5) was prepared and filtered through a 2 nm filter and autoclaved. Cas9 mRNA, sgRNAs, and ssODNs were diluted and mixed in MIB to the working concentrations of 100 ng/µL, 50 ng/µL each, and 50 ng/µL, respectively. Delivery was via pronuclear injection into 1‐cell stage C57BL/6NTac embryos. Injected embryos were re‐implanted in CD1 pseudo‐pregnant females. Host females were allowed to litter and rear F_0_ progeny.

Hair cell‐specific conditional clarin‐2 knockout mice were obtained by crossing *Clrn2* ^ex2fl/fl^ mice with *Myo15‐cre* ^+/ki^ mice. Primary auditory neuron specific clarin‐2 knockout mice were obtained by crossing *Clrn2* ^ex2fl/fl^ mice with *Bhlhb5‐cre* ^+/ki^ mice.

To generate clarin‐1/clarin‐2 double total knockout mice, *Clrn1* ^−/−^ mice were crossed with *Clrn2* ^−/−^ mice, resulting in *Clrn1* ^−/−^ *Clrn2* ^−/−^ mice. Primary auditory neuron‐specific double clarin‐1/clarin‐2 conditional knockout mice were obtained through crossings of *Clrn1* ^fl/fl^ *Bhlhb5‐cre* ^+/ki^ mice with *Clrn2* ^fl/fl^ *Bhlhb5‐cre* ^+/ki^ mice.

Generally, mice were analyzed at P21, except where otherwise stated. This time‐point was chosen for several reasons: to assess mature hair cells and primary auditory neurons without the risk of cellular loss, which begins in clarin‐mutant mice around P30, and to better integrate and correlate the data from the physiological and RNA‐sequencing data with immunostainings. Wild‐type littermates were used for all conditional clarin‐mutant mice experiments; however, separate wild‐type breedings were used for total knockout mice. Each experiment was carried out with at least 2 separate litters per genotype with appropriate wild‐type controls.

### 4.3 Genotyping

To identify wild‐type, homozygous, and heterozygous mice, toe biopsies were collected between P5‐8 and genotyped. DNA was extracted from each sample using the REDExtract‐N‐AMP Tissue PCR kit (Sigma‐Aldrich, XNAT). A polymerase chain reaction (PCR) was then performed using mouse line‐specific primers (Table S5) and REDExtract‐N‐AMP PCR reaction mix (Sigma–Aldrich, R4775), using an Eppendorf Mastercycler Nexus—PCR Thermal Cycler. The PCR products were separated by electrophoresis on a 1.5% agarose gel in 1X TAE (Tris, Acetate, EDTA) buffer. The electrophoresis gel was visualized using ultraviolet light and imaged with a Vilber E‐box.

The nonsense mutation (p.Trp4*) in *Clrn2* in Clrn2*^−/−^* and *Clrn1* ^−/−^ *Clrn2* ^−/−^ mice was confirmed via sequencing after PCR amplification (Eurofins Genomics).

### 4.4 Audiological Testing

Auditory tests were performed on mice anesthetized with a mixture of Ketamine (100 mg/kg) and Xylazine (2 mg/kg). Core temperatures of anesthetized mice were maintained at 37°C with a regulated heating pad, and eyes were protected with Ocry‐Gel (tvm, 3338–951).

The Distortion product otoacoustic emissions (DPOAE) at a frequency 2f1‐f2 were recorded in response to two primary tones of similar energy levels, f1 and f2, with f2/f1 = 1.20 using OtoPhyLab (Echodia). Frequency f2 was swept at 1/10th octave steps from 8 to 32 kHz, with primary tone levels decreasing from 70 to 20 dB SPL in 5 dB steps. DPOAE threshold was plotted against frequency f2. The DPOAE threshold was defined as the weakest stimulus producing a DPOAE significantly above the background noise.

The Auditory Brainstem Responses (ABR) in response to calibrated short tone bursts in the 5–40 kHz range (repetition rate 17/s) were derived by the synchronous averaging of electroencephalograms recorded between subcutaneous stainless‐steel electrodes at the vertex and ipsilateral mastoid, with the help of a standard digital averaging system (CED1401+). Three hundred responses to the tone bursts were averaged at different sound pressure levels (dB SPL), descending by 10 dB from 100 to 10 dB. ABR threshold was defined as the smallest tone‐burst level giving rise to at least one repeatable wave above background noise levels. At suprathreshold levels (90 dB SPL), wave I amplitudes and latencies were measured using a custom‐designed MATLAB interface.

### 4.5 Mechanoelectrical Transduction Current Recordings

All recordings were performed at cochlear positions lying 20%–40% from the cochlear apex of P6‐P8 mice as previously described [72]. The inner ears of mice were carefully dissected to reveal the cochlea for electrophysiological measurements, and placed in an extracellular solution (146 mm NaCl, 5.8 mm KCl, 1.5 mm CaCl_2_, 0.7 mm NaH_2_PO_4_, 2 mm of sodium pyruvate, 10 mm Glucose, and 10 mm HEPES, pH 7.4), and OHCs were whole‐cell voltage clamped at at −80 mV and 20–25°C. Borosilicate pipettes (1–2 MΩ) were filled with a solution of 130 mm KCl, 10 mm NaCl, 2.5 mm MgCl_2_, 1 mm EGTA, 5 mm ATP potassium, 0.5 mm GTP, and 5 mm HEPES, pH 7.4. No correction was made for liquid junction potential. OHC hair bundles were displaced mechanically using a rigid fire‐polished glass rod, 2–3 µm in diameter. MET currents of OHCs were recorded using the EPC‐10 patch‐clamp amplifier and patchmaster software (Heka Elektronik, Lambrecht, Germany). A fast voltage amplifier (ENV800; Jena piezosystem) was used to secure the probe to an electric actuator (PA8/12; Jena Piezo System) for the test. The reaction resonance of the actuator was restricted to a small bandpass of 5 kHz by the Bessel four‐pole filter (model 3362; Krohn‐Kite), which was accompanied by the relaxing signal of the EPC‐10 amplifier. MET current responses were averaged over five consecutive stimulations.

### 4.6 Whole‐Cell Patch Clamp Recordings of BK Currents

Whole‐cell patch‐clamp recordings of BK currents were made in IHCs from ex vivo whole‐mount preparation of organs of Corti freshly dissected from P18‐P24 mice. BK recordings were performed in IHCs issued from the 20%–40% normalized distance from the apex, an area coding for frequencies ranging from 8 to 16 kHz, by using an EPC10 amplifier controlled by Patchmaster pulse software (HEKA Elektronik, Germany). Recording pipettes were filled with a KCl‐based intracellular solution containing 158 mm KCl, 2 mm MgCl_2_, 1.1 mm EGTA, 5 mm HEPES, and 3.05 mm KOH, pH 7.20. Patch pipettes were pulled with a micropipette Puller P‐97 Flaming/Brown (Sutter Instrument, Novato, CA, USA) and fire‐polished with a Microforge MF‐830 (Narishige, Japan) to obtain a resistance range from 3 to 5 MΩ). Voltage drop across series resistance (Rs 2–5 MΩ) and leak current were electronically compensated. The organs of Corti were incubated in an extracellular perilymph‐like solution containing 135 mm NaCl, 5.8 mm KCl, 1.3 mm CaCl_2_, 0.9 mm MgCl_2_, 0.7 mm NaH_2_PO_4_, 5.6 mm Glucose, 2 mm Na pyruvate, 10 mm HEPES, pH 7.4, 305 mOsm. All experiments were performed at room temperature (RT, 22–24°C).

### 4.7 Exocytosis Measurement

Real‐time capacitance measurements (C_m_) were performed using the Lock‐in amplifier Patchmaster software (HEKA) by applying a 1 kHz command sine wave (amplitude 20 mV) at holding potential (−80 mV) before and after the pulse experiment, as previously described [16, 13]. Patch pipettes were filled with an intracellular cesium‐based solution (145 mm CsCl, 1 mm MgCl_2_, 5 mm HEPES, 1 mm EGTA, 20 mm tetraethylammonium chloride, 2 mm ATP, and 0.3 mm GTP, pH 7.2, 300 mOsm). The organs of Corti from P18‐P24 mice were bathed in an extracellular solution (135 mm NaCl, 5.8 mm KCl, 5 mm CaCl_2_, 0.9 mm MgCl_2_, 0.7 mm NaH_2_PO_4_, 5.6 mm glucose, 2 mm Na pyruvate, and 10 mm HEPES, pH 7.4, 305 mOsm). The extracellular solution was complemented with 0.25 µm of apamin (Latoxan, L8407) and 1 µm of XE‐991 (Tocris Bioscience, 2000) to block SK channels and KCNQ4 channels, respectively. All experiments were performed at RT. Because recording conditions can greatly influence capacitance measurements, only IHC patch‐clamp recordings with low series resistance below 10 mΩ and a maximum leak current of 25 pA (at V_h_ = −80 mV) were considered in the present study.

### 4.8 Bimolecular Fluorescence Complementation (BiFC) Assay

The BiFC co‐expression vector containing the sequences coding for VN173 (amino acids 1–172) or VC155 (amino acids 155–238) fragments of mVenus protein with the appropriate linker sequences (SGLRSSA and SGLRSPAS) was designed with an auto‐cleavable F2A peptide sequence [73,74]. The amino acid sequences of mouse MET genes were placed into pTwistCMV plasmids (Twist Bioscience) as BiFC co‐expression vectors. *Clrn1* and *Clrn2* were tagged N‐terminally with VN173, and the MET and Tip‐link proteins were tagged with VC155 N‐terminally or C‐terminally.

HeLa cells (ATCC) were cultivated in DMEM‐F‐12 supplemented with GlutaMAX (Invitrogen, 31331028) with 10% fetal bovine serum, 50 U/ml penicillin, and 50 mg/ml streptomycin (Invitrogen, 15140122) at 37°C and 5% CO_2_ in T‐75 flasks. HeLa cells were seeded at ∼0.1 × 10^6^ cells into 12‐well plates containing 15 mm diameter round coverslips in each well. The next day, when HeLa cells reached a confluence of 60%–80%, they were transfected with Lipofectamine 3000 reagent (Invitrogen, L3000008) according to the instructions of the manufacturer with co‐expression vectors. 24 h post‐transfection, cells were fixed in 4% PFA for 15 min at RT. Cells were stained for DAPI and mounted with FluorSave Reagent (EMD Millipore, 345789).

### 4.9 Inner Ear Tissue Preparation

Mice were injected intraperitoneally with a lethal cocktail of Ketamine and Xylazine, and inner ears were collected in 1X Phosphate Buffer solution (PBS) (Sigma‐Aldrich, P3813) for either whole organ of Corti immunostaining or inner ear collection for cryosectioning. Generally, extraneous tissue from the inner ears was removed in PBS, and the round and oval windows of the cochlea were cleared, a hole was placed at the apex of the cochlea, and the posterior semicircular canal was broken to allow perfusion of 4% paraformaldehyde (PFA) diluted in PBS (Electron Microscopy Sciences, 15714). Inner ears were fixed for 1 h at RT.

### 4.10 Organ of Corti Immunostaining and Whole Mount

After fixation in 4% PFA, inner ears were diluted into 1% PFA, and then the organ of Corti was microdissected from the cochlea in PBS. Whole organs of Corti were then washed in PBS for 5 min and blocked and permeabilized using 20% normal goat serum (NGS) (Gibco, 16210072), 0.3% triton in PBS for 1 h at RT. Primary antibodies were diluted in a 1% Bovine serum albumin (BSA)‐PBS solution at various concentrations (Table S6) and incubated with the organs of Corti overnight at 4°C. The following day organs of Corti were washed 3 times with PBS and then incubated with secondary antibodies (Table S7) diluted in 1% BSA‐PBS for 1 h at RT. Organs of Corti were then washed once with PBS and then incubated with DAPI (4′,6‐diamidino‐2‐phénylindole, dichlorhydrate) (Invitrogen, D1306) diluted to 1:1000 in PBS for 15 min at RT. Organs of Corti were then washed three times in PBS.

For GluR2 staining, organs of Corti were blocked in 20% horse serum (HS) (Gibco, 16050122), 0.4% triton in PBS, and both primary and secondary antibodies were diluted into 1% HS, 0.1% triton in PBS. Primary antibodies were incubated overnight at 37°C, while secondary antibodies were incubated for 2 h at 37°C. For PSD95, inner ears were extracted in ice‐cold PBS and perfused by injection through the round and oval windows with ice‐cold Zamboni fixative (Newcomer Supply 1459A) and then submerged in ice‐cold Zamboni while rocking on ice for 10 min. Inner ears were then washed 3 times with ice‐cold PBS. The organ of Corti was then microdissected out of the cochlea in ice‐cold PBS for blocking and permeabilization for 2 h at RT in 1X blocking solution (16% NGS + 450 mm NaCl + 20 mm phosphate buffer (Na_2_HP0_4_.2H_2_0, NaH2PO4.H2O) + 0.3% Triton‐X). Primary antibodies were diluted in 1X blocking solution, and organs of Corti were incubated overnight at 4°C. The next day organs of Corti were washed 3 times in wash buffer (2 mm phosphate buffer, 450 mm NaCl, H20). Secondary antibodies were diluted in 1X blocking solution, and the organs of Corti were incubated for 1 h at RT. Organs of Corti were then washed once with wash buffer and incubated with DAPI in PBS for 15 min at RT. They were then washed 3 times in PBS at RT and mounted on superfrost slides (ThermoFisher, 12392098) in FluorSave Reagent (EMD Millipore, 345789), and 1.5 borosilicate coverglass (VWR, 631‐0125).

### 4.11 Inner Ear Cryosectioning and Immunostaining

After fixation in 4% PFA, inner ears were washed 3 times in PBS. To decalcify the bone of the inner ear and allow for cryosectioning of the underlying tissue, inner ears were placed in 0.35 m EDTA‐PBS, pH 7.5, at 4°C. Inner ears were then washed 3 times in PBS and post‐fixed for 1 h in 4% PFA at RT. Inner ears were then washed 3 times in PBS and placed in 20% sucrose‐PBS overnight at 4°C. Inner ears were then mounted in OCT cryomax (Epredia, 6769006) in cryomolds (Tissue‐Tek, 4566) and frozen in a bath of liquid nitrogen. Frozen inner ear blocks were then stored at −80°C until cryosectioning.

Cryosections from inner ear blocks were cut using the Epredia Cryostat CryoStar NX50. Serial 14 µm inner ear cryosections were collected at −20°C on SuperFrost Plus slides (ThermoFisher, 22037246). Slides were dried at RT for 2 h before being stored at −20°C for immunostaining or RNAscope.

For immunostaining, inner ear sections were blocked and permeabilized in 20% NGS, 0.3% triton in PBS for 1 h at RT in a humid chamber. Inner ear sections were then incubated with primary antibodies (Table S6) diluted in 1% BSA‐PBS overnight at 4°C in a humid chamber. The next day, slides were washed 3 times in PBS and then incubated with secondary antibodies (Table S7) diluted in 1% BSA‐PBS for 1 h at RT in a humid chamber. Slides were then washed once in PBS and then incubated with DAPI for 15 min in a humid chamber at RT. Slides were then washed three times in PBS and mounted with FluorSave Reagent and 1.5 borosilicate coverglass (VWR, 631‐0138).

### 4.12 RNAscope on Inner Ear Cryosections

RNAscope was performed using RNAscope Multiplex Fluorescent V2 Assay (Advanced Cell Diagnostic, 323270), following manufacturer instructions with slight modification. Slides were washed in PBS while rocking to remove excess OCT and then post‐fixed in 4% PFA for 15 min at RT. They were washed twice with milliQ water and then dehydrated in successively increasing concentrations of ethanol and then dried for 30 min at 40°C. Inner ear sections were blocked and permeabilized in RNAscope Hydrogen Peroxide for 10 min at RT and then washed twice with milliQ water. Slides were then incubated with RNAscope Protease Plus for 20 min at 40°C, then washed twice in milliQ water at RT. Inner ear sections were then hybridized with target probes (*Clrn1* in C1, *Clrn2* in C3, or *Lypd1* in C2) by incubation for 2 h at 40°C. They were then washed with saline sodium citrate solution, pH 7. Slides were then washed in 1X RNAscope washing buffer and post‐fixed in 4% PFA for 10 min at RT. The probe signal was successively amplified using RNAscope Multiplex Fl v2 Amp1‐3 for 30 min each at 40°C. After final amplifying hybridization, probe signals were developed using RNAscope Multiplex Fl v2 HRP‐C1, ‐C2, or ‐C3 by incubation for 15 min at 40°C. Slides were then washed twice with RNAscope washing buffer at RT. Inner ear sections were then incubated with Vivid Fluorophore 570 or 650 diluted 1:1000 in Tyramide Signal amplification (TSA) buffer for 30 min at 40°C. Slides were then washed twice in RNAscope washing buffer, then incubated in RNAscope Multiplex Fl v2 HRP Blocker for 15 min at 40°C. Slides were then washed twice in RNAscope washing buffer at RT. After RNAscope was complete, immunostaining for parvalbumin was performed as described above.

### 4.13 Microscopy and Image Analysis

Images were acquired using confocal microscopes: Airyscan/LSM‐900 (Zeiss, Germany), LSM 700 (Zeiss, Germany), and Leica Stellaris 5 (Leica, Germany). The objectives used were 63× NA 1.4 apochromatic plane oil immersion objective and a 20× NA 0.8 apochromatic plane lens without immersion. Images were analyzed using Fiji (Open Source). For hair bundle analyses, whole‐mount F‐actin–labeled bundles were scored manually by blinded observers using maximum‐intensity projections. Hair bundles were classified as: (1) normal—intact V‐ or U‐shaped bundles with preserved staircase architecture; (2) misaligned—abnormal orientation or disrupted row pattern; or (3) fragmented—broken, truncated, splayed, or collapsed stereocilia. For each genotype, OHCs and IHCs from ≥3 mice were analyzed, and data were aggregated as mean ± SD.

Ribbon, synaptic, BK channel cluster counts were performed manually using the multi‐point tool, while GluR2 and PSD95 morphology were analyzed using area, shape descriptors, perimeter, and Feret's diameter, limited to threshold using the analyze particles tool. Feret's diameter of GluR2 patches was delineated into small, medium, and large bins based on the mean and distribution of the GluR2 patches in the wild‐type mice. Primary auditory neuron counts were performed manually using the multi‐point tool, and counts were normalized to the area of the Rosenthal canal, measured using the freehand selection tool.

For BiFC fluorescence quantification, NIS‐6 Software (Nikon, Japan) was used to count the cells for each construct; images containing between 350 and 950 cells were analyzed.

### 4.14 Whole Organ of Corti RNA‐Sequencing and Quantitative Real‐Time PCR

At post‐natal day 21, wild‐type, *Clrn1* ^−/−^, *Clrn2* ^−/−^, and *Clrn1* ^−/−^ *Clrn2* ^−/−^ mice were injected intraperitoneally with a lethal cocktail of Ketamine and Xylazine, and inner ears were collected in ice‐cold 1X HBSS (ThermoFisher, 24020117). Organs of Corti were quickly dissected out of the cochlea, removing the stria vascularis and modulus, and collected in RNAs‐free 2 mL micro tubes to be immediately flash frozen in liquid nitrogen. Organs of Corti were collected in triplicate and kept at −80°C until RNA extraction.

RNA extraction and purification were performed using the NucleoSpin RNA Mini kit for RNA purification (Macherey‐Nagel, 740955.50), following manufacturer instructions with slight modifications in quantities of reagents used. RNA samples were measured with NanoDrop to assess RNA concentration and determine if there was any protein or ethanol contamination. 2 µg RNA samples were sent for RNA‐sequencing at Integragen.

RNA libraries were prepared with NEBNext Ultra II Directional RNA Library Prep Kit for Illumina protocol according to the supplier recommendations. Briefly, the key stages of the protocol are successively: the purification of Poly‐A containing mRNA molecules using poly‐T oligo attached magnetic beads from 100 ng total RNA (with the Magnetic mRNA Isolation Kit from NEB), a fragmentation using divalent cations under elevated temperature to obtain approximately 300 bp pieces, double strand cDNA synthesis and finally Illumina adapters ligation and cDNA library amplification by PCR for sequencing. Sequencing is then carried out on Paired End 100b reads of Illumina NovaSeq 6000. Image analysis and base calling were performed using Illumina Real Time Analysis (3.4.4) with default parameters.

Gene expression was quantified using Integragen's Galileo software. STAR was used to obtain the number of reads associated with each gene in the Gencode vM24 annotation (restricted to protein‐coding genes, antisense, and lincRNAs). Raw counts for each sample were imported into R statistical software using the Bioconductor DESeq2 package. The extracted count matrix was normalized for library size and coding length of genes to compute FPKM expression levels.

Unsupervised analysis of data used the Bioconductor edgeR package to import raw counts into R statistical software and compute normalized log2 CPM (counts per million of mapped reads) using the TMM (weighted trimmed mean of M‐values) as a normalization procedure. The normalized expression matrix from the 1000 most variant genes (based on standard deviation) was used to classify the samples according to their gene expression patterns using principal component analysis (PCA). PCA was performed by FactoMineR::PCA function with “ncp = 10, scale.unit = FALSE” parameters.

The Bioconductor edgeR package was used to import raw counts into R statistical software for differential expression analysis of clarin mutant mice relative to wild‐type controls. Differential expression analysis was performed using the Bioconductor limma package and the voom transformation. To improve the statistical power of the analysis, only genes expressed in at least one sample (FPKM ≥ 0.1) were considered. A q‐value threshold of ≤ 0.05 and a minimum fold change of 1.5 were used to define differentially expressed genes.

Differential expression analysis tables were exported from Galileo, and each gene was annotated with the known biological process, molecular function, and cell compartment gene ontology (GO) terms. Based on GO terms, genes were categorized into 8 main pathways: cationic flux, synaptic function, neuronal function & differentiation, mitochondrial function (including glucose metabolism and oxidative stress), endocytosis & exocytosis, lipid homeostasis, actin & cytoskeletal organization, and inflammation. Genes implicated in hearing were also noted. Genes in all these categories were significantly dysregulated in each differential expression analysis; however, genes of interest were selected based on the significant genes found in *Clrn1* ^−/−^ *Clrn2* ^−/−^ relative to wild‐type. Heat maps were generated using the log_2_Fold change of each gene of interest.

For quantitative real‐time PCR, cDNAs were prepared from 1 µg of total RNA in triplicate using the SuperScript IV VILO Master Mix. RT‐PCR products were digested with RNaseH (Thermo Fisher, 18021–071). Quantitative RT‐PCR was performed with GoTaq qPCR Master Mix containing BRYT Green Dye (Promega, A6002) using specific primers for each of 14 genes related to mechanoelectrical transduction and for the glyceraldehyde‐3‐phosphate dehydrogenase (*Gapdh*) as an internal control (Table S8). Quantitative real‐time PCR reactions were performed using the Quant Studio 6 Flex Real‐Time PCR System (Applied Biosystems). The thermocycling conditions were 50°C for 2 min, followed by 95°C for 10 min, and then 40 cycles of 95°C for 15 s and 60°C for 1 min. Relative levels of target transcripts were determined by the comparative cycle threshold (CT) method. The relative copy number for each target transcript was calculated as 2^−ΔΔCT^. Transcription levels in the organ of Corti were compared, for each gene, between *Clrn1* ^−/−^ *Clrn2* ^−/−^ and wild‐type mice, in two‐way ANOVA with Šidák correction for multiple comparisons.

### 4.15 Scanning and Transmission Electron Microscopy

For scanning electron microscopy, peri‐auricular regions were quickly extracted from P8, P21, and P60 mice, and a small hole was made in the apex of the cochlea. Samples were fixed in 2.5% glutaraldehyde (EMS, 16220) diluted in 0.1 m sodium cacodylate (Sigma–Aldrich, C4945)

(pH 7.4) for 2 h at RT before an overnight decalcification at 4°C in 0.35 m EDTA diluted in 0.75% glutaraldehyde, followed by a 2 h postfixation in 2.5% glutaraldehyde at RT. After three washes in PBS, the organs of Corti were micro‐dissected, washed in 0.1 m sodium cacodylate, and then processed with the OTOTO protocol consisting of three incubations of 30 min at RT with 1% tetroxide osmium diluted in 0.1 m sodium cacodylate, alternated with two incubations of 15 min at RT with 0.1 m thiocarbohydrazide (Sigma–Aldrich, 223220). Each of these five steps was separated by six washes in ultrapure water. Samples were then dehydrated through a graded ethanol series (50%, 70%, 85%, 95%, 100%, 5 min each) followed by 15 min in hexamethyldisilazane and air drying. Samples were then mounted on carbon film glued to aluminum stubs (Oxford Instruments) and coated with gold/palladium using a Quorum Q150R S sputter coater (Quorum Technologies). Scanning images were acquired with a JEOL IT700HR scanning electron microscope. For homogeneous visualization, a light blue overlay was uniformly applied across all the scanning electron microscopy micrographs.

For transmission electron microscopy, 1‐month‐old mice were injected intraperitoneally with a lethal cocktail of Ketamine and Xylazine, and their inner ears were collected in PBS. Extraneous tissue from the inner ears was removed in PBS, and the round and oval windows of the cochlea were cleared; a hole was placed at the apex of the cochlea. Inner ears were fixed through round and oval window perfusion of 2% PFA/2.5% glutaraldehyde solution and then submerged in 2% PFA/2.5% glutaraldehyde while slowly rocking for 2 h at RT. Inner ears were then washed twice in PBS and placed in 0.35 m EDTA‐PBS to decalcify at 4°C. Decalcified inner ears were washed with water and then post‐fixed in 1% Osmium Tetroxide (EMS, 19150) overnight at 4°C, samples were protected from light. After washing twice with water, the inner ears were successively dehydrated using increasing concentrations of acetone in 1‐h incubations. Samples were embedded in Spurr resin (EMS), followed by polymerization for 48 h at 60°C. Ultrathin sections (70 nm thick) were obtained with an ultramicrotome (Reichert Ultracut E) equipped with a diamond knife (Diatome). The sections were mounted on copper grids coated with collodion. Sections for morphological analysis were contrast‐stained with uranyl acetate and lead citrate for 15 min each. The ultrathin sections were observed under a JEM‐1400 transmission electron microscope (Jeol) at 80 kV.

### 4.16 Statistical Analyses

Data are expressed as mean + standard error of the mean (SEM). The number (*n*) in figures and text corresponds to the number of mice. Mice in each group come from at least two independent litters. For whole organ of Corti analyses, at least 30 inner hair cells were quantified per mouse. For inner ear cryosections, at least 3 spiral ganglia along the length of the middle turn were analyzed per mouse.

All statistical analyses were performed with GraphPad software. One‐way and Two‐way ANOVAs were performed, with Tukey's multiple comparisons. Statistical significance is indicated in the figures as follows: * *p* <0.05; **; *p* <0.01; ***; *p* <0,001; ****; *p* <0,0001. Differences were considered statistically significant if *p* < 0.05. Non‐significant comparisons are not indicated.

## Author Contributions

S.D. and A.E. are joint senior authors and co‐supervised the work. S.D. conceived the project. S.D. and M.W. designed the experiments and analyzed all the data. M.W., A.Y.S., and M.H. carried out histological/immunohistochemical and RNAscope studies. M.W., A.Y.S., S.L.G., and S.N. managed mouse lines. M.W., A.Y.S., and S.D. carried out ABR and DPOAEs recordings. D.D. and S.C. carried out in vivo electrophysiology and exocytosis measurements. P.P., S.L.G., and S.V. performed scanning electron microscopy. M.S. and K.Y.Y. performed BiFC experiments. A.L. carried out in vivo electrophysiology and patch‐clamp mechanoelectrical transmission current measurements. S.D., M.W, M.T., and N.T. carried out and conducted the transmission electron microscopy work. M.W. and S.D. collected the organs of Corti and RNA‐extraction for RNA‐seq. A.Y.S. and S.D. performed the quantitative PCR experiments. E.W., M.W., and S.D. processed and analyzed the raw data of RNA‐Seq. M.R.B. generated and supplied the *Clrn2* ^fl/fl^ mouse line. M.W., S.D., and A.E. wrote the paper. All the authors read and approved the manuscript before submission.

## Conflicts of Interest

The authors declare no conflicts of interest.

## Ethics Statement

Institut Pasteur animal facilities are accredited by the French Ministry of Agriculture for experimentation on live mice (accreditation 75‐15‐01, issued on September sixth, 2013) in application of the French and European regulations on the care and protection of laboratory animals (EC Directive 2010/63, French Law 2013–118, February sixth, 2013). Animal testing was carried out with the approval of the Institut Pasteur ethics committee (Institut Pasteur‐DAP180039 and DAP240030).

## Supporting information




**Supporting File 1**: advs73883‐sup‐0001‐SuppMat.docx.

**Supporting File 2**: advs73883‐sup‐0002‐Tables.zip.

**Supporting File 3**: advs73883‐sup‐0003‐SuppMat.pdf.

## Data Availability

All data needed to evaluate the conclusions in the paper are present in the paper and/or the Supporting Information. The data can be provided by the corresponding authors pending scientific review and a completed material transfer agreement. The accession number for the RNA‐sequencing data reported in this paper is deposited in GEO: GSE312253.
