# Supplementary material for: Compensatory Interplay Between Clarin‐1 and Clarin‐2 Deafness‐Associated Proteins Governs Phenotypic Variability in Hearing
Source: Adv Sci (Weinh). 2026 Jan 22;13(20):e21853. doi: 10.1002/advs.202521853 (PMC13067776; doi:10.1002/advs.202521853)
Supplement: Supplementary file 1 — Supporting File 1: advs73883‐sup‐0001‐SuppMat.docx. [file ADVS-13-e21853-s002.docx]

*Supplementary information Research Article (advs.202521853R2)*

**Compensatory interplay between clarin-1 and clarin-2 deafness-associated proteins govern phenotypic variability in hearing**

Maureen Wentling^1,2^, Aïda Yakhlef Sanchez^1,3^, Nicolas Thelen^4^, Müge Senarisoy^1^, Maria Hogg^1^, Steven Condamine^5^, Andrea Lelli^6^, Emilia Wysocka^1^, Pranav Patni^1,2^, Sandrine Vitry^1^, Kerem Yasin Yildizhan^1^, Sébastien Le Gal^1^, Sylvie Nouaille^1^, Michael R. Bowl^7^, Marc Thiry^3^, Didier Dulon^5^, Sedigheh Delmaghani^1*^, Aziz El-Amraoui^1*^

^1^ Université Paris Cité, Institut Pasteur, AP-HP, INSERM U1335, CNRS, Fondation Pour l’Audition, Institut de l'Audition, IHU reConnect, Progressive Sensory Disorders, Pathophysiology and Therapy, F-75012 Paris, France.

^2^ Sorbonne Université, Collège Doctoral ED515, F-75005 Paris, France.

^3^ Sorbonne Université, Collège Doctoral ED3C, F-75005 Paris, France.

^4^ Cellular and Tissular Biology, GIGA-Neurosciences, University of Liège, Liège, Belgium.

^5^ Institut de l’Audition and Université de Bordeaux, Laboratoire de Neurophysiologie de la Synapse Auditive, Bordeaux Neurocampus, 33076 Bordeaux, France.

^6^ Université Paris Cité, Institut Pasteur, AP-HP, INSERM, CNRS, Fondation Pour l'Audition, Institut de l'Audition, IHU reConnect, Auditory Therapies Innovation Laboratory, Paris, F-75012, France.

^7^UCL Ear Institute, University College London, London, UK.

* Co-senior and Corresponding authors: Dr Sedigheh Delmaghani & Dr Aziz El Amraoui; Unit Progressive Sensory Disorders, Pathophysiology and Therapy; Institut Pasteur, Institut de l’Audition, Paris, France; [sedigheh.delmaghani@pasteur.fr](mailto:sedigheh.delmaghani@pasteur.fr) ; [aziz.el-amraoui@pasteur.fr](mailto:aziz.el-amraoui@pasteur.fr)

**
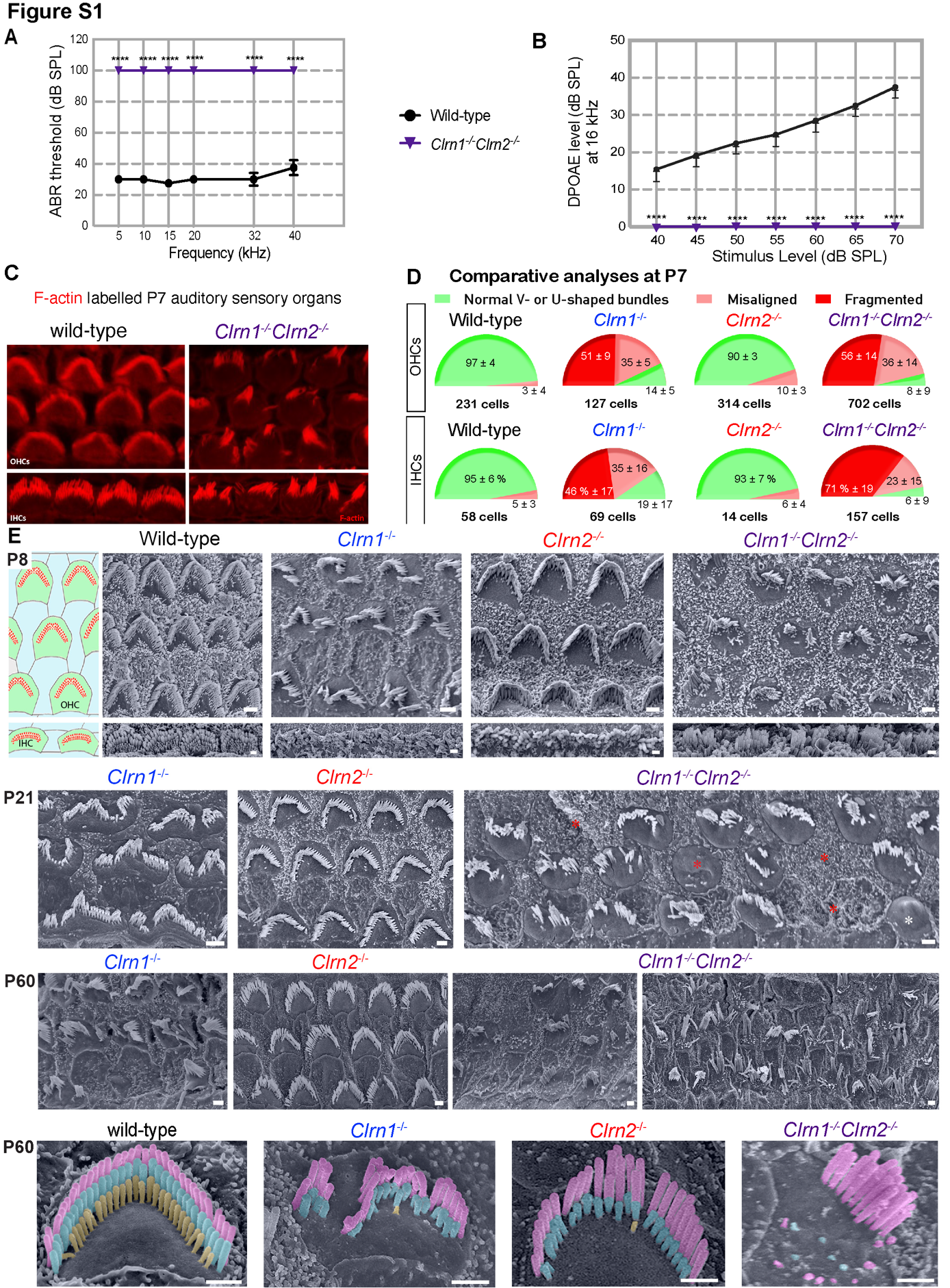
Figure S1. Auditory Thresholds in *Clrn1*^-/-^*Clrn2*^-/-^ mice.** **A)** ABR thresholds in wild-type mice (black trace) and *Clrn1*^-/-^*Clrn2*^-/-^ (purple trace) at P21. **B)** DPOAEs amplitudes at 16 kHz in wild-type and *Clrn1*^-/-^*Clrn2*^-/-^ mice at P21. Wild-type and *Clrn1*^-/-^*Clrn2*^-/-^ n=5; mean ± SEM ; T-tests were performed at each frequency tested. **C)** F-actin–labeled stereocilia morphology in cochlear hair cells of WT and *Clrn*1^-/-^*Clrn2*^-/-^ mice at P7. Whole-mount phalloidin staining shows the sharply defined V-shaped OHC bundles and U-shaped IHC bundles characteristic of wild-type cochleae. In *Clrn1*^-/-^*Clrn2*^-/-^ mice, stereocilia exhibit severe cytoskeletal disorganization, appearing fragmented, splayed, truncated, or collapsed, with frequent loss of the coherent staircase architecture normally present in both OHCs and IHCs. **D)** Quantification of OHC and IHC bundle morphology across clarin genotypes at P7. The pie shart diagrams summarize the proportion of hair bundles classified as normal (green), misaligned (light red), or fragmented (red) based on phalloidin-labeled stereocilia. In OHCs, wild-type cochleae (97 ± 4%, *n* = 231) and *Clrn2*^-/-^ mice (90 ± 3%, *n* = 314) retain predominantly normal V-shaped bundles, whereas *Clrn1*^-/-^ OHCs show marked disruption, with only 14 ± 5% normal bundles and most exhibiting misalignment (35 ± 5%) or fragmentation (51 ± 9%). The defect is most extreme in *Clrn1*^-/-^*Clrn2*^-/-^ OHCs, where only 8 ± 9% (*n* = 702) remain intact. IHCs exhibit a similar genotype-dependent gradient: *Clrn2*^-/-^ IHCs maintain normal U-shaped morphology, whereas *Clrn1*^-/-^ and *Clrn1*^-/-^*Clrn2*^-/-^ cochleae show substantial deterioration. In *Clrn1*^-/-^*Clrn2*^-/-^, only 6–19% of IHCs retain recognizable bundles, with severe fragmentation predominating (71 ± 19%, *n* = 157). For each genotype, OHCs and IHCs from ≥3 mice were analyzed, and data were aggregated as mean ± SD. **E)** Scanning electron microscopy images of OHCs at P8, P21, and P60 from wild-type, *Clrn1*^-/-^, *Clrn2*^-/-^, and *Clrn1*^-/-^*Clrn2*^-/-^ mice, illustrating the prgression of Hair bundle abnormalities over time. Asterisks (red) indicate positions of lost OHC bundles and/or lost hair cells. Stereocilia are pseudo-colored to indicate tallest (pink), middle (teal), and short (yellow) rows.

**Figure S2. Transcriptomic analysis reveals dysregulation of hearing-related genes in clarin-mutant mice.** **A)** RNA-seq analysis performed on whole organs of Corti at P21. Principal component analysis (PCA) of wild-type (grey), *Clrn1*^-/-^ (blue), *Clrn2*^-/-^ (red), and *Clrn1*^-/-^*Clrn2*^-/-^ (purple) samples. Volcano plots depict significantly dysregulated genes in each mutant genotype relative to wild-type. Log₂(fold change) is shown on the x-axis and −log₁₀(q-value) on the y-axis. Genes were considered significantly differentially expressed at q < 0.05 with an absolute fold change ≥ 1.5; significantly upregulated genes are shown in red and downregulated genes in green. **B)** Heat map of hearing-related genes defined by Gene Ontology terms associated with sensory perception of sound, cochlear or inner ear development, and inner ear morphogenesis. Log₂(fold change) values are shown, with upregulated genes in red and downregulated genes in green. All genes displayed were significantly dysregulated (q ≤ 0.05) in *Clrn1*^-/-^*Clrn2*^-/-^ mice relative to wild-type. **C)** Quantitative real-time PCR validation of selected dysregulated genes in P21 organs of Corti from *Clrn1*^-/-^*Clrn2*^-/-^ mice relative to wild-type controls (n = 3 per group). Data were analyzed using two-way ANOVA with Šidák correction for multiple comparisons; values are shown as mean ± SEM.

**Figure S3. Transcriptional dysregulation of cation flux–related genes in clarin-deficient cochleae.** Heat maps depict genes involved in cation homeostasis that are significantly dysregulated in clarin-mutant mice, based on RNA-seq analysis of whole organs of Corti at postnatal day 21 (P21). Upregulated genes are shown on the left and downregulated genes on the right. Genes are ordered by decreasing absolute fold change and grouped according to their primary functional association, with calcium-handling genes listed first, followed by potassium- and sodium-related genes. Color intensity represents log₂ fold change relative to wild-type controls, with upregulation shown in red and downregulation in green. Only genes reaching statistical significance (adjusted q ≤ 0.05) in *Clrn1*^-/-^*Clrn2*^-/-^ mice compared with wild-type are displayed. These data highlight coordinated alterations in ionic homeostasis pathways associated with combined clarin deficiency.

**Figure S4. Auditory function in hair cell-specific clarin-knockout mice.** **A, B)** Auditory brainstem response (ABR) thresholds measured at postnatal day 21 (P21) and 1 month of age in wild-type (black trace), *Clrn1*^fl/fl^*Myo15-cre*^+/ki^ (blue trace), *Clrn2*^fl/fl^*Myo15-cre*^+/ki^ (red trace), and *Clrn1*^fl/fl^*Clrn2*^fl/fl^*Myo15-cre*^+/ki^ (purple trace) mice. **C)** Distortion product otoacoustic emission (DPOAE) amplitudes of wild-type (black trace), *Clrn1*^fl/fl^*Myo15-cre*^+/ki^ (blue trace), *Clrn2*^fl/fl^*Myo15-cre*^+/ki^ (red trace), and *Clrn1*^fl/fl^*Clrn2*^fl/fl^*Myo15-cre*^+/ki^ (purple trace) mice at P21 for 16 kHz. **D)** DPOAE thresholds at 16kHz for P21 wild-type, *Clrn1*^fl/fl^*Myo15-cre*^+/ki^, *Clrn2*^fl/fl^*Myo15-cre*^+/ki^ mice. Wild-type n=20 (P21) and n= 14 (1 month), *Clrn1*^fl/fl^*Myo15-cre*^+/ki^ n=7 (P21) and n=5 (1 month), *Clrn2*^fl/fl^*Myo15-cre*^+/ki^ n=14 (P21) and n=9 (1 month), and *Clrn1^fl/fl^Clrn2*^fl/fl^*Myo15-cre*^+/ki^ n=5 (P21). Data are presented as mean ± SEM. **A-C**: t-tests were performed at each frequency tested. **D**: data were analyzed using one-way ANOVA with Tukey’s multiple comparisons test.

**Figure S5. Transcriptomic alterations in synaptic signaling and organization in clarin-mutant mice.** Heat maps showing genes involved in glutamatergic synaptic transmission (left), synaptic organization and assembly (middle), and synaptic endocytosis and exocytosis (right) that are differentially expressed in clarin-mutant mice. Log₂ fold change relative to wild-type is indicated, with upregulated genes shown in red and downregulated genes in green. All displayed genes meet the significance threshold (q ≤ 0.05) in *Clrn1*^-/-^*Clrn2*^-/-^ mice compared with wild-type controls. RNA-seq analysis was performed on whole organs of Corti at postnatal day 21 (P21).

**Figure S6. Expression of Clarin-1 and Clarin-2 in primary auditory neurons.** RNAscope in situ hybridization reveals expression of Clrn1 (red, upper panels) and Clrn2 (red, lower panels) in primary auditory neurons of P21 wild-type mice. Neurons are identified by parvalbumin immunostaining (green). Images are shown from the middle (left) and basal (right) turns of the cochlea. Scale bar, 50 µm.

**Figure S7. Mitochondrial abnormalities in primary auditory neurons of clarin-mutant mice.**

**A)** Representative transmission electron microscopy (TEM) images showing mitochondrial morphology in primary auditory neurons from wild-type, *Clrn1*^-/-^, *Clrn1*^fl/fl^*Myo15-cre^+/ki^*, *Clrn2*^-/-^, *Clrn2*^fl/fl^*Myo15-cre*^+/ki^, *Clrn1*^-/-^*Clrn2*^-/-^, *Clrn1*^fl/fl^*Clrn2*^fl/fl^*Myo15-cre*^+/ki^, and *Clrn1*^fl/fl^*Clrn2*^fl/fl^*Bhlhb5-cre*^+/ki^ mice at 1 month. Yellow arrows indicate mitochondria containing vacuoles, and white arrows indicate mitochondria with disrupted cristae**. B)** Quantification of degenerating mitochondria in primary auditory neurons at 1 month across genotypes. n = 4 mice per genotype. Data are presented as mean ± SEM and analyzed using one-way ANOVA with Tukey’s multiple comparisons test.

**Figure S8. Dysregulation of metabolic pathways in clarin-mutant mice.** Heat maps showing genes involved in mitochondrial function (left), glucose metabolism and glycolysis (middle), and oxidative stress responses (right) in clarin-mutant mice. Log₂ fold-change values are indicated, with upregulated genes shown in red and downregulated genes shown in green. All displayed genes are significantly dysregulated (q ≤ 0.05) in *Clrn1*^-/-^*Clrn2*^-/-^ mice relative to wild-type controls. RNA-seq analysis was performed on whole organs of Corti at P21.

**Figure S9. Bhlhb5-Cre expression is specific, stable, and restricted to primary auditory neurons and type I subtypes.** **A)** Parvalbumin immunostaining (green) and TdTomato autofluorescence (red) of primary auditory neurons in *Bhlhb5-cre*^+/ki^*Rosa-TdTomato*^+/ki^ mice at P21 (left panel) and 6 months (right panel) at the middle turn (top panel) and basal turn (bottom panel) of the cochlea. Parvalbumin (green) immunostaining and TdTomato (red) autofluorescence of hair cells and afferent fibers in *Bhlhb5-cre*^+/ki^*Rosa-TdTomato*^+/ki^ mice at P21 in the middle turn of the cochlea. Percent of TdTomato and parvalbumin double-positive neurons relative to overall parvalbumin-positive neurons. P21, 3 months, and 6 months n= 4; mean ± SEM. **B)** Calretinin (green, top panel), Calbindin (green, middle panel) and Parvalbumin (blue) immunostaining, *Lypd1* (green, bottom panel) RNAscope labeling and TdTomato (red) autofluorescence of primary auditory neurons in *Bhlhb5-cre*^+/ki^*Rosa-TdTomato*^+/ki^ mice at 1.5 months in the middle turn of the cochlea. Bar charts represent the percentage of TdTomato and subtype marker double-positive neurons relative to the total subtype specific neuronal marker population, and the percentage of parvalbumin and subtype marker double-positive neurons relative to the total parvalbumin-positive neurons. Each neuronal subtype n= 4; mean ± SEM.
