# Supplementary material for: Compensatory Interplay Between Clarin‐1 and Clarin‐2 Deafness‐Associated Proteins Governs Phenotypic Variability in Hearing
Source: Adv Sci (Weinh). 2026 Jan 22;13(20):e21853. doi: 10.1002/advs.202521853 (PMC13067776; doi:10.1002/advs.202521853)
Supplement: Supplementary file 2 — Supporting File 2: advs73883‐sup‐0002‐Tables.zip. [file ADVS-13-e21853-s003.zip › advs202521853_Table S8.docx]

**Table S8.** Primers used for qRT-PCR analysis of dysregulated MET-related hearing genes in *Clrn1*^-/-^*Clrn2*^-/-^ mice.

| **Gene name** | **Accession number** | **Forward primer** | **Reverse primer** |
| --- | --- | --- | --- |
| *Ceacam16* (CEA Cell Adhesion Molecule 16, Tectorial Membrane Component) | NM_001033419.2 | CTACGGACACATGCAGGTCTATG | CAACTGGCAGAGCGTCACCATT |
| *Otog* (Otogelin) | NM_013624.2 | ACCTGTGAGTGGCGCTACGATG | GTGACTTCATCCAGGACCTTAGG |
| *Otogl* (Otogelin-like) | NM_001177567.2 | TCATTGGCTCTTGTTTCCTTG | TTCTTCCGAGTCATCGTATTTT |
| *Tectb* (Tectorin-B) | NM_009348.4 | GGTCCATGAAGGTGGCTATTACC | TGTCGTTGGACACGATGTGGCT |
| *Col11a2* (Collagen Type XI Alpha 2 Chain) | NM_009926.3 | AGGACGGCTTTCCTGGGTTCAA | TGTAGGTCCAGTGCGCCCTTTT |
| *Espn* (Espin) | NM_207687.3 | TCGTTCCTCGTCTTCTACTGGC | CCGCCTTTATCTCAGCCAGAAG |
| *Espnl* (Espin Like) | NM_001033292.4 | AGGTGATGGTGCGGAAGCTACA | CTCCAAGTTGCCTGTTCTGCAG |
| *Pcdh15* (Protocadherin-15) | NM_001142746.1 | CTCTGGAAGTGGTCCTTGCCAA | GCGAACATCCTTGCGTCTTCAG |
| *Tmc1* (Transmembrane Channel Like 1) | NM_028953.3 | TCATTGGCGACTTCCTCAGAGC | GCCTTGGTTGAAGATCAGAGCG |
| *USH1C* (Harmonin) | NM_153677.2 | GCAGATTTCCTCTGCAGAGAATG | GGATGTCATCCAGGTCAGTGGT |
| *Strc* (Stereocilin) | NM_080459.2 | GTAGAACAGGGTCTGCTGGAATG | CCTCCAGATGAACGCAACACTC |
| *Diaph1* (Diaphanous Related Formin 1) | NM_001305980.2 | ATCCTGGAGGTGAATGAGGCTG | CCACACCAAACTGCTCTGACTC |
| *Ptprq* (Protein Tyrosine Phosphatase Receptor Type Q) | NM_001081432.2 | GTATGGCTGACGTGGAGTCCAA | GTCCAGCAAGTTTGGCATCAGTC |
| *Pdzd7* (PDZ domian containing 7) | NM_001195265.1 | CTGAGGGACATCAGGAGCGTGG | AGCAGAAGGCCCCACTGCCCTG |
| *Gapdh* (Glyceraldehyde-3-Phosphate Dehydrogenase ; internal control) | NM_008084.4 | CATCACTGCCACCCAGAAGACTG | ATGCCAGTGAGCTTCCCGTTCAG |
