# Supplementary material for: Compensatory Interplay Between Clarin‐1 and Clarin‐2 Deafness‐Associated Proteins Governs Phenotypic Variability in Hearing
Source: Adv Sci (Weinh). 2026 Jan 22;13(20):e21853. doi: 10.1002/advs.202521853 (PMC13067776; doi:10.1002/advs.202521853)
Supplement: Supplementary file 2 — Supporting File 2: advs73883‐sup‐0002‐Tables.zip. [file ADVS-13-e21853-s003.zip › advs202521853_Table S7.docx]

**Table S7.** Secondary antibodies used for immunostaining.

| **Antibody** | **Reference** |
| --- | --- |
| Goat anti Mouse IgG1 AlexaFluor 594 | Invitrogen, A21121 |
| Goat anti Mouse IgG2a AlexaFluor 594 | Invitrogen, A21135 |
| Goat anti Mouse Atto 550 | Sigma, 43394 |
| Goat anti Mouse Atto 647 | Sigma, 50185 |
| Goat anti Rabbit Atto 488 | Sigma, 18772 |
| Goat anti Rabbit Atto 550 | Sigma, 43328 |
| Goat anti Rabbit Atto 647 | Sigma, 40839 |
| Donkey anti Chicken FITC | ThermoFisher, SA1-72000 |
| Phalloidin Atto 565 | Sigma, 94072 |
