# Supplementary material for: Compensatory Interplay Between Clarin‐1 and Clarin‐2 Deafness‐Associated Proteins Governs Phenotypic Variability in Hearing
Source: Adv Sci (Weinh). 2026 Jan 22;13(20):e21853. doi: 10.1002/advs.202521853 (PMC13067776; doi:10.1002/advs.202521853)
Supplement: Supplementary file 2 — Supporting File 2: advs73883‐sup‐0002‐Tables.zip. [file ADVS-13-e21853-s003.zip › advs202521853_Table S6.docx]

**Table S6.** Primary antibodies used for immunostaining.

| **Antibody** | **Species** | **Reference** |
| --- | --- | --- |
| Myo7a | Rabbit | Proteus, 25-6790 |
| Ribeye | Mouse IgG1 | Bd Biosences, 612044 |
| GluR2 | Mouse IgG2a | Millipore, MAB397 |
| PSD95 | Rabbit | Abcam, ab238135 |
| PMCA2 | Rabbit | Thermofisher, PA1-915 |
| BK Channel | Rabbit | Alamone Labs, APC-021 |
| Parvalbumin | Mouse | Sigma, SAB4200545 |
| NF200 | Chicken | Millipore, AB5539 |
| Calretinin | Rabbit | Swant, CR7697 |
| Calbindin | Rabbit | Cell Signaling, 13176S |
