# Supplementary material for: Compensatory Interplay Between Clarin‐1 and Clarin‐2 Deafness‐Associated Proteins Governs Phenotypic Variability in Hearing
Source: Adv Sci (Weinh). 2026 Jan 22;13(20):e21853. doi: 10.1002/advs.202521853 (PMC13067776; doi:10.1002/advs.202521853)
Supplement: Supplementary file 2 — Supporting File 2: advs73883‐sup‐0002‐Tables.zip. [file ADVS-13-e21853-s003.zip › advs202521853_Table S5.docx]

**Table S5.** Primers used for genotyping.

| **Mouse line** | **PCR** | **Foward Primer** | **Reverse Primer** |
| --- | --- | --- | --- |
| *Clrn1*^-/-/^*Clrn1*^fl/fl^ | PCR1  PCR2 | CCACATGTCACCACCATCCTCAC  GGGTCTGACCTCCAGGTCAGGGCAC | CTTTGAAGGTGCCCAGCAATTCAGG CTTTGAAGGTGCCCAGCAATTCAGG |
| *Clrn2*^-/-^ | PCR1 | TGAGGGAGGATGAACTAGCG | GATGAGAAACTGAGTAGAGATGCCA |
| *Clrn2*^fl/fl^ | PCR1  PCR2  PCR3 | GGCAATAACATTTACTCTGCCAAG ATCCGGGGGTACCGCGTCGAG ACTAGCACCAAAGGCAAGCAAGCATAG | CTCATTTTTTCTCTTCCAGACTG ACTGATGGCGAGCTCAGACC CTGAGGCATATAAAGTTTGCATGAC |
| *Myo15-cre* | PCR1  PCR2 | AGGGACCTGACTCCACTTTGGG AGGGACCTGACTCCACTTTGGG | GGAACTGACCTTTCTTAGAGATCTTGGG TGGTGCACAGTCAGCAGGTTGG |
| *Bhlhb5-cre* | PCR1  PCR2 | CCTGACTCTCCAGCCCAGGTG CATGGTGAGCCTTAGCAGCCAG | GATCAGCGGGCTCGAAACAG AGCTGGCCCAAATGTTGCTG |
| *Bhlhb5-cre*  *RosaTd-Tomato* | PCR1  PCR2 | AAGGGAGCTGCAGTGGAGTA GGCATTAAAGCAGCGTATCC | CCGAAAATCTGTGGGAAGTC CTGTTCCTGTACGGCATGG |
