# Supplementary material for: Compensatory Interplay Between Clarin‐1 and Clarin‐2 Deafness‐Associated Proteins Governs Phenotypic Variability in Hearing
Source: Adv Sci (Weinh). 2026 Jan 22;13(20):e21853. doi: 10.1002/advs.202521853 (PMC13067776; doi:10.1002/advs.202521853)
Supplement: Supplementary file 2 — Supporting File 2: advs73883‐sup‐0002‐Tables.zip. [file ADVS-13-e21853-s003.zip › advs202521853_Table S4.docx]

**Table S4.** Sequences of oligonucleotides utilised for the generation of the *Clrn2*^ex2fl^ allele.

| **Oligonucleotide name** | **Sequence** |
| --- | --- |
| *Clrn2* CRISPR sgRNA protospacer, 5’_1 (PAM) | ACTCTGCCAAGCCAATGCCT (TGG) |
| *Clrn2* CRISPR sgRNA protospacer, 5’_2 (PAM) | AGCCAATGCCTTGGGCTTAT (GGG) |
| *Clrn2* CRISPR sgRNA protospacer, 3’_1 (PAM) | AGGGCACTTTTATTGCAGTC (TGG) |
| *Clrn2* CRISPR sgRNA protospacer, 3’_2 (PAM) | AGACTCAAAAGGAAGCTAAA (GGG) |
| *Clrn2* CRISPR genotyping primer (forward) | ACGAGCTCACTCAACCCCTAA |
| *Clrn2* CRISPR genotyping primer (reverse) | TGAAGGTCCGCCTTTGACCA |
| *Clrn2* CRISPR copy counting ddPCR primer (forward) | GCTCTGGTCAGCATGGGTT |
| *Clrn2* CRISPR copy counting ddPCR primer (reverse) | GGGCCATTGACTGCTCTGTA |
| CRISPR copy counting ddPCR probe | TTGCTATTCTCAACATCATTCAGGTCCC |
