# Supplementary material for: Compensatory Interplay Between Clarin‐1 and Clarin‐2 Deafness‐Associated Proteins Governs Phenotypic Variability in Hearing
Source: Adv Sci (Weinh). 2026 Jan 22;13(20):e21853. doi: 10.1002/advs.202521853 (PMC13067776; doi:10.1002/advs.202521853)
Supplement: Supplementary file 3 — Supporting File 3: advs73883‐sup‐0003‐SuppMat.pdf. [file ADVS-13-e21853-s001.pdf]

Raw and processed (light filter overlay) SEM micrographs in Figure 1A and supplementary Figure S1E

Figure 1A

Raw, unprocessed scanning electron microscopy (SEM) micrographs

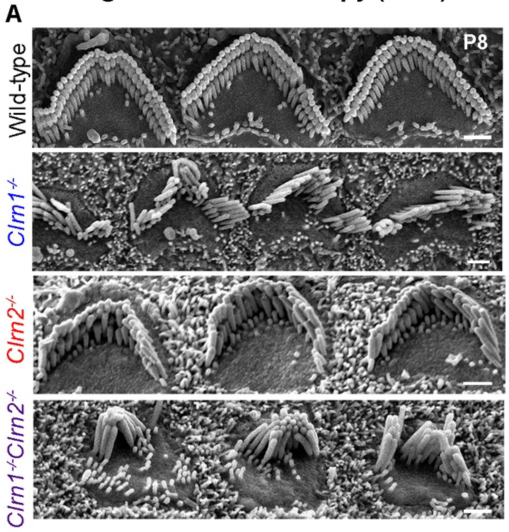

SEM images with uniform light-blue overlay

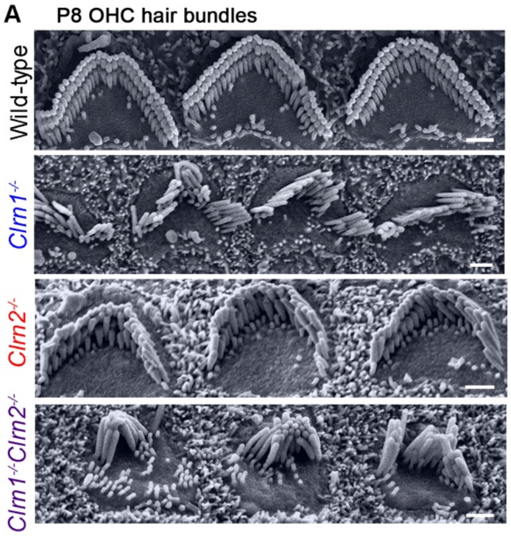

Supplementary Figure S1E

Raw, unprocessed SEM micrographs

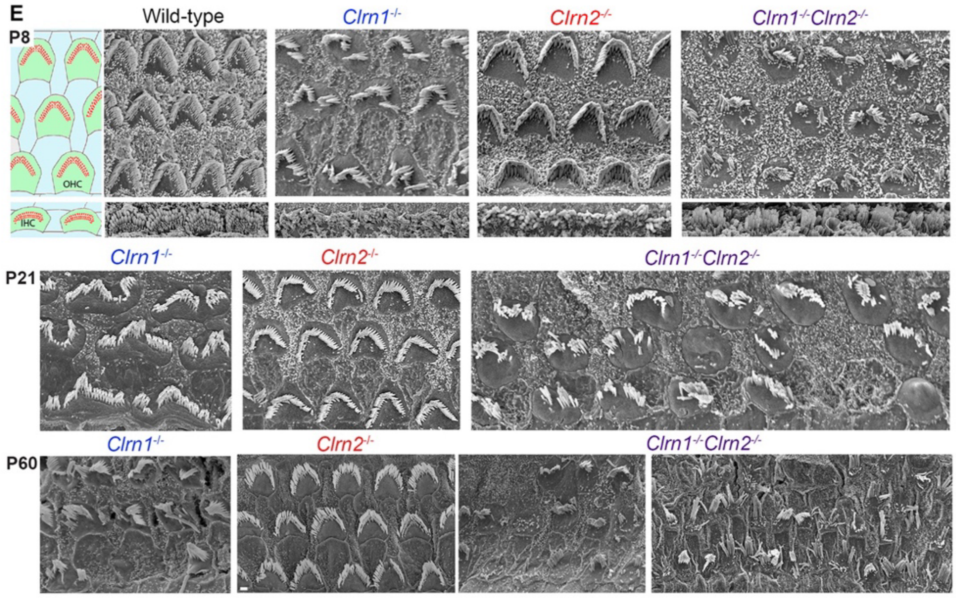

SEM images with uniform light-blue overlay

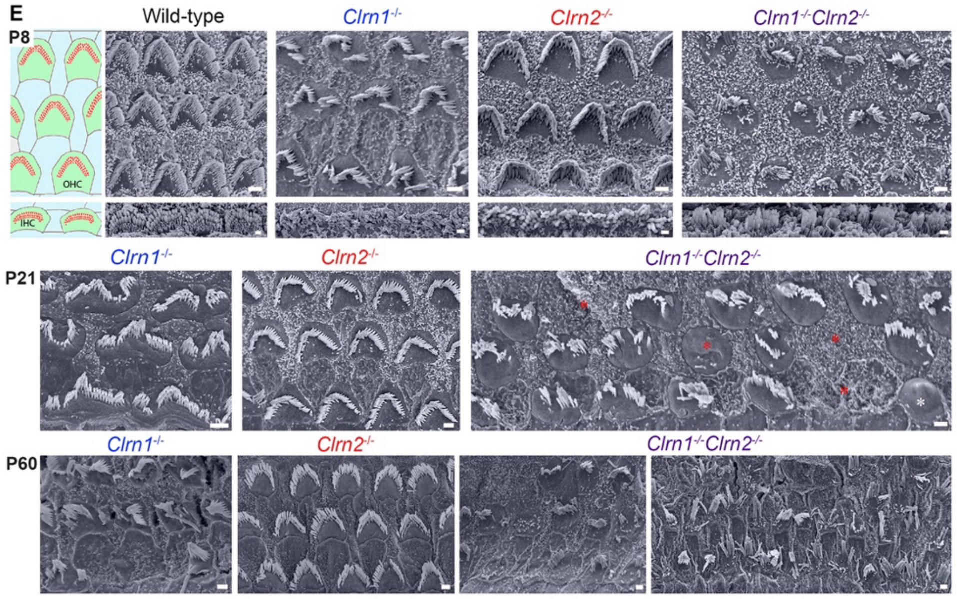

For homogeneous visualization, a light blue overlay was uniformly applied across all the scanning electron microscopy micrographs. Unprocessed raw images, identical except for the absence of this overlay, are provided as Supporting Information.

Raw and Cropped SEM Images of OHC Stereocilia Bundles used in Figure S1E

Raw, unprocessed scanning electron microscopy (SEM) micrographs

Cropped and colorized outer hair cell (OHC) hair bundles shown in Supplementary Figure S1E

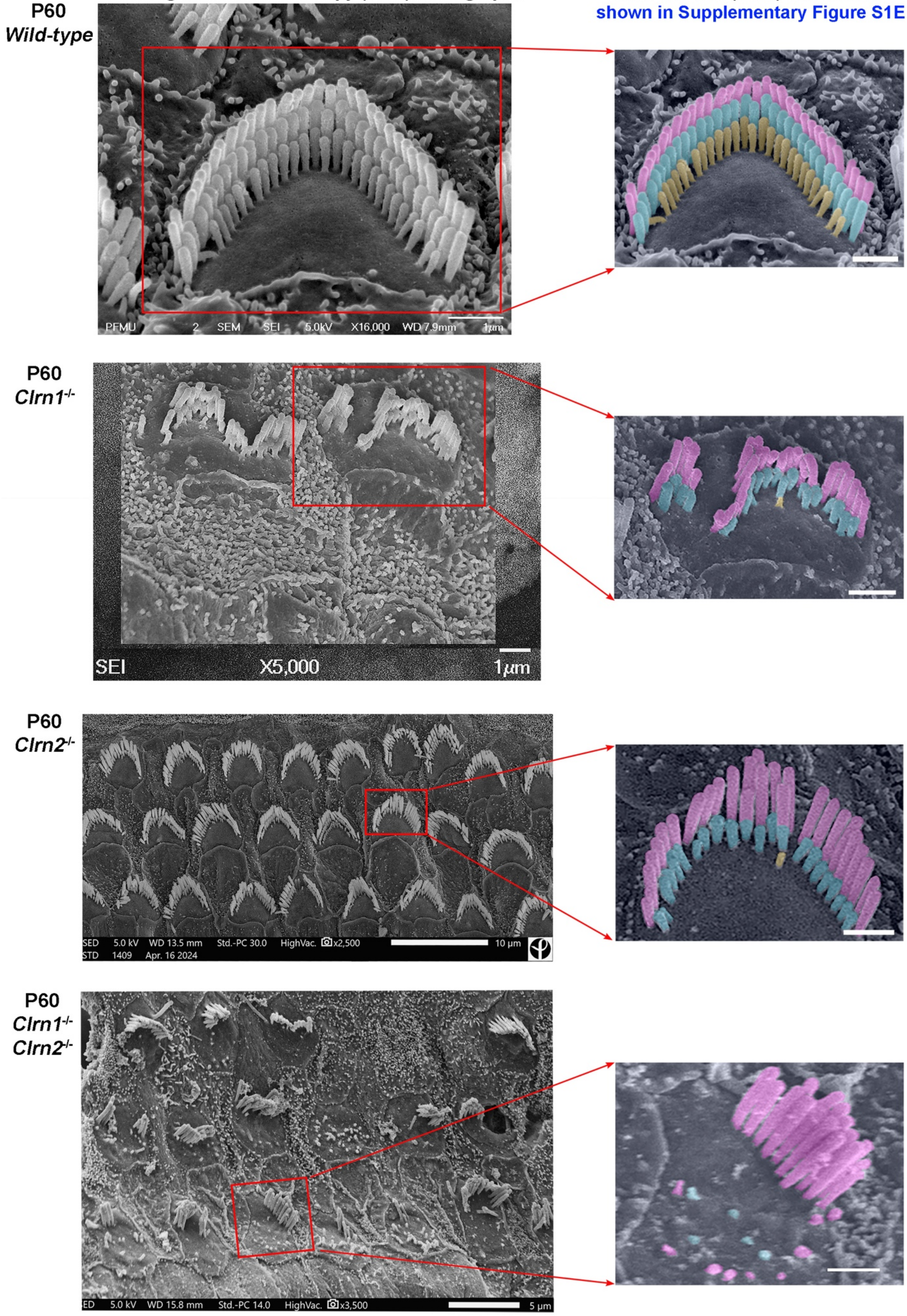

Left panels show the full, raw, unprocessed scanning electron microscopy (SEM) images that served as the source data for the zoomed OHC hair-bundle images presented in Supplementary Figure 1E. **Right panels** display the cropped regions (red squares in left panels) highlighting individual stereocilia rows, pseudo-colored to indicate the tallest row (purple), middle row (blue), and shortest row (yellow).
